# The Complexity of Drug Development: Translational Value and Limitations of Computational ADMET Assays Applied to Approved Anticancer Drugs

**DOI:** 10.3390/ph19060840

**Published:** 2026-05-28

**Authors:** Mirela Nicolov, Adina Octavia Dușe, Elena-Daniela Jurj, Daiana Colibășanu, Adrian Voicu, Claudia Watz, Mirela Voicu, Lucreția Udrescu

**Affiliations:** 1 Center for Drug Data Analysis, Cheminformatics, and the Internet of Medical Things, “Victor Babeș” University of Medicine and Pharmacy Timișoara, Eftimie Murgu Square, No. 2, 300041 Timișoara, Romania; mirelanicolov@umft.ro (M.N.); daniela.adam@umft.ro (E.-D.J.); daiana.handa@umft.ro (D.C.); adrian.voicu@umft.ro (A.V.); farcas.claudia@umft.ro (C.W.); 2 Department I–Physical Chemistry and Pharmaceutical Physics, “Victor Babeș” University of Medicine and Pharmacy Timișoara, Eftimie Murgu Square, No. 2, 300041 Timișoara, Romania; 3 Department of Rehabilitation, Physical Medicine and Rheumatology, Faculty of Medicine, “Victor Babeș” University of Medicine and Pharmacy Timișoara, Eftimie Murgu Square, No. 2, 300041 Timișoara, Romania; duse.adina@umft.ro; 4 Transdisciplinary Research Center in Medical Rehabilitation, Balneology and Rheumatology, “Victor Babeș” University of Medicine and Pharmacy Timișoara, Eftimie Murgu Square, No. 2, 300041 Timișoara, Romania; 5 Departament II–Pharmaceutical Chemistry, “Victor Babeș” University of Medicine and Pharmacy Timișoara, Eftimie Murgu Square, No. 2, 300041 Timișoara, Romania; 6 Department II–Pharmacology, Physiology and Physiopathology, “Victor Babeș” University of Medicine and Pharmacy Timișoara, Eftimie Murgu Square, No. 2, 300041 Timișoara, Romania; 7 OncoGen Center for Gene and Cellular Therapies in the Treatment of Cancer, “Pius Brînzeu” County Clinical Emergency Hospital, 300723 Timișoara, Romania; 8 Research Center for Experimental Pharmacology and Drug Design (X-Pharm Design), “Victor Babeș” University of Medicine and Pharmacy Timișoara, Eftimie Murgu Square, No. 2, 300041 Timișoara, Romania; 9 Department I–Clinical Pharmacy and Drug Analysis, “Victor Babeș” University of Medicine and Pharmacy Timișoara, Eftimie Murgu Square, No. 2, 300041 Timișoara, Romania

**Keywords:** ADMET, FAF-Drugs4, SwissADME, anticancer drugs, pharmacokinetics, toxicity prediction, drug development

## Abstract

**Background:** This study evaluates the translational relevance of free computer-assisted ADMET platforms in drug discovery by comparing SwissADME and FAF-Drugs4 predictions with regulatory and curated reference data for approved anticancer drugs. **Methods:** Fourteen approved anticancer agents representing diverse chemical classes were analyzed using SwissADME and FAF-Drugs4. We compared predicted physicochemical, pharmacokinetic, and toxicity-related properties with information extracted from FDA- and EMA-approved product information, DrugBank, and PubChem. We evaluated the concordance for oral absorption, permeability, metabolic stability, and toxicity-related trends. **Results:** The platforms showed good concordance for broad descriptor-driven properties, particularly oral suitability and physicochemical trends. Strong agreement was observed for intravenously administered taxanes, which displayed unfavorable oral drug characteristics, and for several orally active small molecules with generally compatible profiles. Partial concordance was observed for compounds such as temozolomide, whose clinical behavior is influenced by factors not fully captured by descriptor-based models. Toxicity outputs were informative as early warning signals, with vandetanib showing the clearest alignment between predicted elevated risk and documented safety concerns. **Conclusions:** Free computational ADMET tools are valuable computer-assisted drug discovery resources for early triage, predictive toxicology, and prioritization of repositioning candidates, but they should complement rigorous experimental and clinical evaluation.

## 1. Introduction

Drug discovery and development are characterized by high costs, lengthy timelines, and significant attrition rates, with many candidate compounds failing due to inadequate pharmacokinetic profiles or unacceptable toxicity. Early assessment of absorption, distribution, metabolism, excretion, and toxicity (ADMET) properties has therefore become integral to rational drug design and candidate prioritization [1,2]. Computational ADMET methods reduce the experimental burden associated with early screening, expedite decision-making, and facilitate the selection of compounds with a greater likelihood of clinical success [1,2,3].

In silico ADMET prediction has transitioned over the past two decades from a supplementary approach to a widely adopted element of preclinical drug discovery, driven by advances in cheminformatics, molecular modeling, machine learning, and the expansion of publicly available pharmacological datasets [1,2,4,5]. Alongside descriptor-based and QSAR approaches, structure-based ADMET prediction has emerged as a complementary technique, although its utility is more limited for broad endpoints such as intestinal absorption and brain permeability [6]. These computational methods are routinely employed to estimate physicochemical properties, drug-likeness, permeability, metabolic stability, and toxicity-related liabilities prior to extensive in vitro and in vivo testing [2,4,5,7]. Their value extends beyond efficiency gains, as they also reduce late-stage failures by enabling earlier identification of liabilities in the development pipeline [1,2].

A broad range of web-based platforms is currently available for computational ADMET assessment, including SwissADME, ADMETlab, ADMET-PrInt, admetSAR, ProTox-II, and FAF-Drugs4 [3,8,9,10,11,12]. Among these, SwissADME is widely used for estimating physicochemical descriptors, pharmacokinetic behavior, drug-likeness, and medicinal chemistry friendliness, whereas FAF-Drugs4 is particularly useful for ADME-tox filtering and early-stage compound triage [3,8]. The increasing accessibility of such tools has made them especially attractive in academic research, and in the early phases of drug development, where rapid and low-cost screening strategies are particularly valuable [2,3,8,13]. Predicting ADMET properties as early as possible in the discovery process can support the selection of higher-quality compounds for experimental evaluation, and several freely accessible tools have been developed specifically for compound filtering, structural alert detection, and early ADMET-oriented prioritization [14]. In addition to numerical descriptors, graphical representations such as egg plots, Golden Triangle plots, and radar-type bioavailability plots have become useful tools for summarizing ADME-related property space and supporting decision-making in medicinal chemistry [15].

Computational ADMET analysis extends its utility beyond de novo drug discovery by facilitating drug repurposing through the identification of approved molecules with pharmacokinetic and safety profiles suitable for new therapeutic indications [16,17,18,19]. This perspective aligns with previous analyses that advocate for early ADME–Tox evaluation as part of a “fail early, fail cheap” strategy and emphasize the importance of integrating computational and experimental ADME–Tox assessments [20]. Network-based computational frameworks have further advanced drug repositioning by revealing pharmacological communities, inferring novel drug properties, and prioritizing candidates for subsequent validation through literature mining or molecular docking [21,22,23]. Approved drugs, having undergone extensive experimental and clinical characterization, can serve as benchmark compounds for assessing the translational relevance of in silico prediction platforms. Comparing predicted and established pharmacological behaviors provides a practical way to evaluate the accuracy of freely accessible ADMET tools in reflecting real-world drug properties.

Anticancer drugs represent an informative benchmark due to their structural diversity and distinct pharmacokinetic and toxicity profiles, including both intravenous cytotoxic agents and oral targeted therapies. This diversity permits evaluation across multiple ADMET-relevant dimensions within a clinically significant compound set. Furthermore, the pharmacokinetic properties and safety liabilities of approved anticancer drugs are well-documented, making them suitable for retrospective comparison.

This study evaluates 14 approved anticancer drugs using SwissADME and FAF-Drugs4, comparing computational predictions with pharmacokinetic and safety data from FDA, EMA, DrugBank, and PubChem. The objective is to assess how well freely accessible computational ADMET tools reproduce established trends in oral absorption, permeability, metabolic stability, and toxicity of approved anticancer drugs. It is hypothesized that these tools will be most concordant for broad descriptor-driven properties, while showing limited agreement for outcomes influenced by formulation, route-specific development strategies, transport mechanisms, or complex toxicity patterns. Beyond the retrospective comparison, the novel contribution of this study is the integration of reference-based concordance assessment, exploratory quantitative endpoint coding, and a practical decision framework for the use of free ADMET prediction tools in early drug discovery.

## 2. Results

### 2.1. Physicochemical Properties

**Figure 1:**
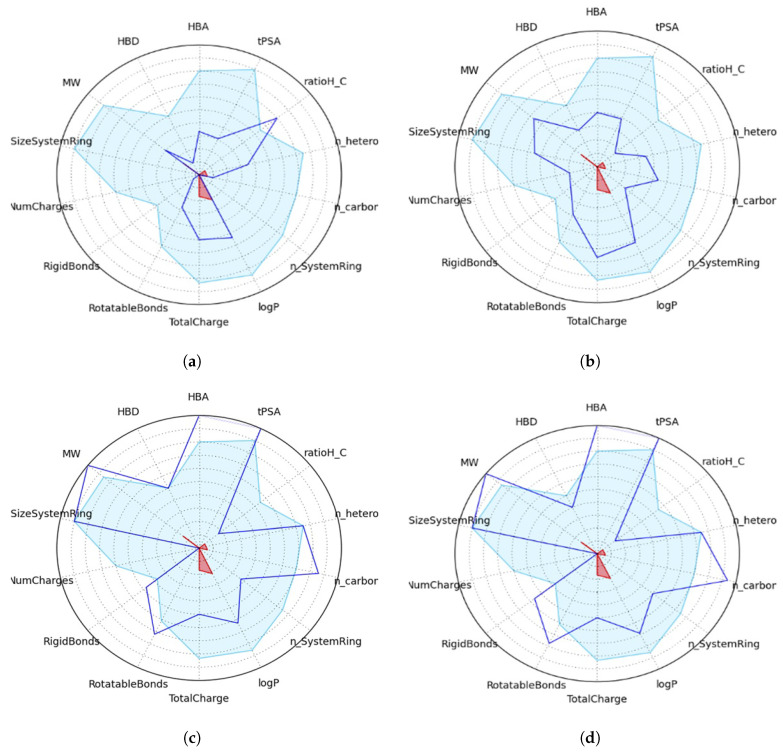
Representative radar plots of the physicochemical properties of selected approved anticancer drugs generated using FAF-Drugs4. Dark blue lines indicate compound profiles, and light blue areas indicate preferred ranges. (**a**) Carmustine and (**b**) sunitinib show profiles that are largely contained within the preferred region, indicating broad compliance with drug-like physicochemical space. (**c**) Docetaxel and (**d**) paclitaxel display multiple deviations from the optimal area.

Figure 1 displays representative radar plots of the physicochemical properties generated using FAF-Drugs4. In each plot, the dark blue line represents the calculated physicochemical profile of an individual compound, while the light blue shaded area denotes the optimal physicochemical space typically associated with drug-like molecules. If the dark blue profile extends beyond the shaded region, the corresponding parameter is outside the preferred range, signifying a deviation from the physicochemical characteristics considered favorable for drug-likeness [8,24].

Carmustine and sunitinib (Figure 1a,b) exhibited physicochemical profiles predominantly within the optimal range, demonstrating broad alignment with the expected physicochemical space of drug-like molecules. In contrast, docetaxel and paclitaxel (Figure 1c,d) exhibited several deviations from the preferred parameters, particularly in molecular weight, topological polar surface area, hydrogen bond acceptor count, and bond-related characteristics. These results indicate that the taxanes represent the most significant physicochemical outliers among the compounds analyzed. The predicted physicochemical descriptors for most anticancer drugs in this study were generally within the optimal drug-like range, suggesting compatibility with the physicochemical space typical of bioactive small molecules. No other compounds deviated from the preferred lipophilicity range as markedly as the high molecular weight taxanes. Radar plots for all compounds analyzed are presented in Figure S1.

### 2.2. Structural Complexity

Molecular complexity parameters, including flexibility (defined as the ratio of rotatable to rigid bonds), fraction of ${\mathrm{sp}}^{3}$ carbon atoms (${\mathrm{Fsp}}^{3}$), number of rigid and rotatable bonds, chiral centers, and ring-related descriptors, are associated with biologically relevant molecular characteristics [25,26]. Nevertheless, universally accepted optimal values for these parameters in compound screening have yet to be established [27].

**Figure 2:**
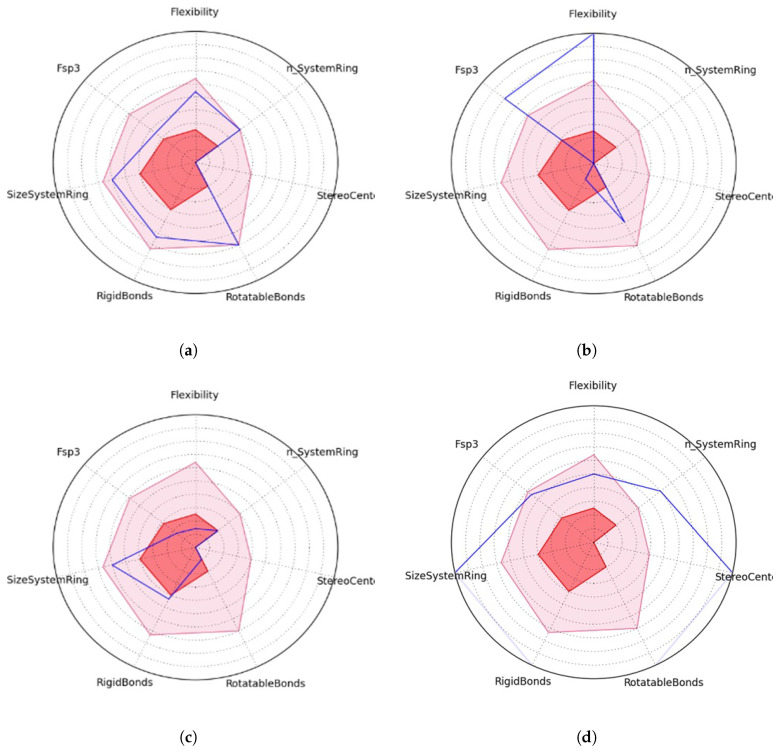
Representative radar plots of the structural complexity profiles of selected approved anticancer drugs generated with FAF-Drugs4. Blue lines indicate compound profiles, and the light red areas indicate preferred ranges. (**a**) Sunitinib shows the closest alignment with the optimal region. (**b**) Carmustine displays increased flexibility and deviation in ${\mathrm{Fsp}}^{3}$. (**c**) Temozolomide shows an extreme flexibility-related value. (**d**) Docetaxel exhibits broader deviations in structural complexity.

Figure 2 presents representative radar plots of the predicted structural complexity profiles of selected approved anticancer drugs. In each plot, the blue line represents the calculated structural complexity profile of an individual compound, whereas the light-red shaded area indicates the preferred range for drug-like molecules. Most investigated anticancer drugs showed at least partial deviation from this optimal region, indicating structural complexity patterns that differ from those typically observed in conventional drug-like compounds.

Sunitinib (Figure 2a) demonstrated the closest overall alignment with the optimal region, reflecting the most balanced structural complexity profile among the compounds analyzed. In contrast, carmustine (Figure 2b) exhibited increased structural flexibility and an ${\mathrm{Fsp}}^{3}$ value outside the preferred range. Temozolomide (Figure 2c) presented an extreme flexibility-related value, with the relevant parameter located within the restricted central region. Docetaxel (Figure 2d) showed broader deviations from the optimal structural complexity space, particularly regarding stereochemical and ring-related descriptors.

Within the full set of investigated compounds, carmustine and lomustine exhibited ${\mathrm{Fsp}}^{3}$ values outside the preferred range, while temozolomide, dabrafenib, trametinib, cabozantinib S-malate, lenvatinib mesylate, and sorafenib demonstrated more pronounced deviations for this descriptor. The remaining structural complexity parameters for most compounds fell within the broader range observed for clinically approved drugs. Radar plots for all investigated compounds are presented in Figure S2.

### 2.3. Permeability and Metabolic Stability Profiles

Permeability and metabolic stability were evaluated using the FAF-Drugs4 Golden Triangle representation, which correlates molecular weight with lipophilicity-related properties. In this framework, compounds situated within the Golden Triangle demonstrate a more optimal balance between permeability and metabolic stability, while those outside this region are more likely to exhibit reduced permeability or increased clearance-related liabilities [28].

**Figure 3:**
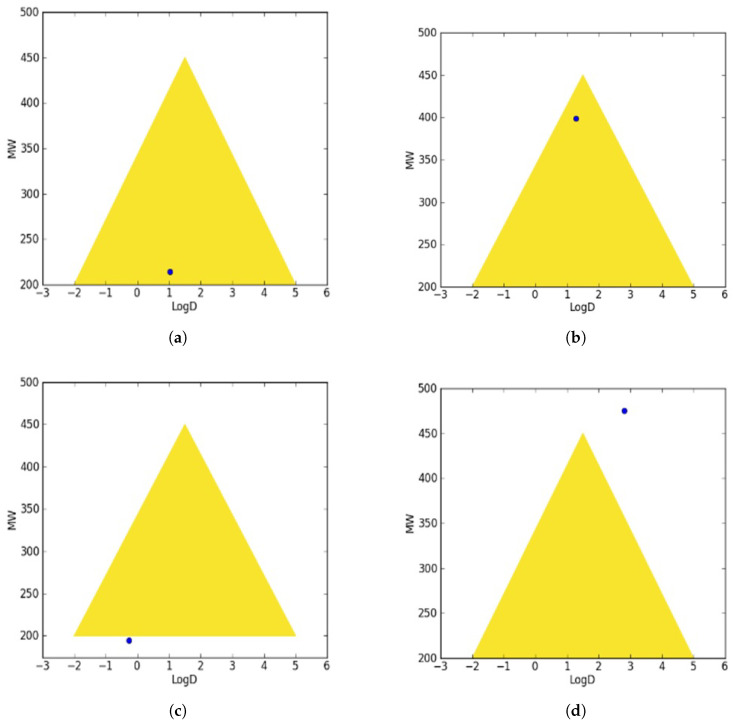
Representative Golden Triangle plots for permeability and metabolic stability of selected approved anticancer drugs generated with FAF-Drugs4. The yellow triangle indicates the optimal region, and the blue dot indicates the analyzed compound. (**a**) Carmustine and (**b**) sunitinib are positioned within the optimal region. (**c**) Temozolomide is located in the lower-logD region, and (**d**) vandetanib is positioned outside the optimal space in the higher molecular weight/higher lipophilicity region.

Figure 3 displays representative Golden Triangle plots for selected approved anticancer drugs. Compounds within the triangle are predicted to possess both favorable permeability and enhanced metabolic stability. Conversely, compounds outside the optimal region tend to fall either into the lower logD space, which correlates with reduced passive permeability, or into the higher molecular weight or higher lipophilicity space, which correlates with diminished metabolic stability.

Carmustine (Figure 3a) and sunitinib (Figure 3b) are positioned within the Golden Triangle, reflecting a physicochemical profile associated with favorable permeability and metabolic stability. In contrast, temozolomide (Figure 3c) occupies the lower logD region, which is indicative of reduced passive permeability. Vandetanib (Figure 3d) is located outside the optimal range, in the higher molecular weight and higher lipophilicity region, suggesting less favorable characteristics for metabolic stability.

Among the investigated compounds, carmustine, lomustine, and sunitinib were positioned within the Golden Triangle, whereas lenvatinib mesylate was situated near its boundary. Dabrafenib, trametinib, vandetanib, erlotinib, cabozantinib S-malate, and sorafenib were found outside the optimal region, specifically in the higher molecular weight and higher lipophilicity space. In contrast, temozolomide and trifluridine were located in the lower logD region, which is associated with reduced passive permeability. Golden Triangle plots for all investigated compounds are presented in Figure S3.

### 2.4. Oral Absorption Profiles

Several physicochemical parameters, including lipophilicity, molecular weight, hydrogen bond donor and acceptor counts, topological polar surface area, and molecular flexibility, influence oral absorption. FAF-Drugs4 visualizes these properties using radar plots to assess the alignment of a compound with physicochemical profiles typically linked to favorable oral absorption.

**Figure 4:**
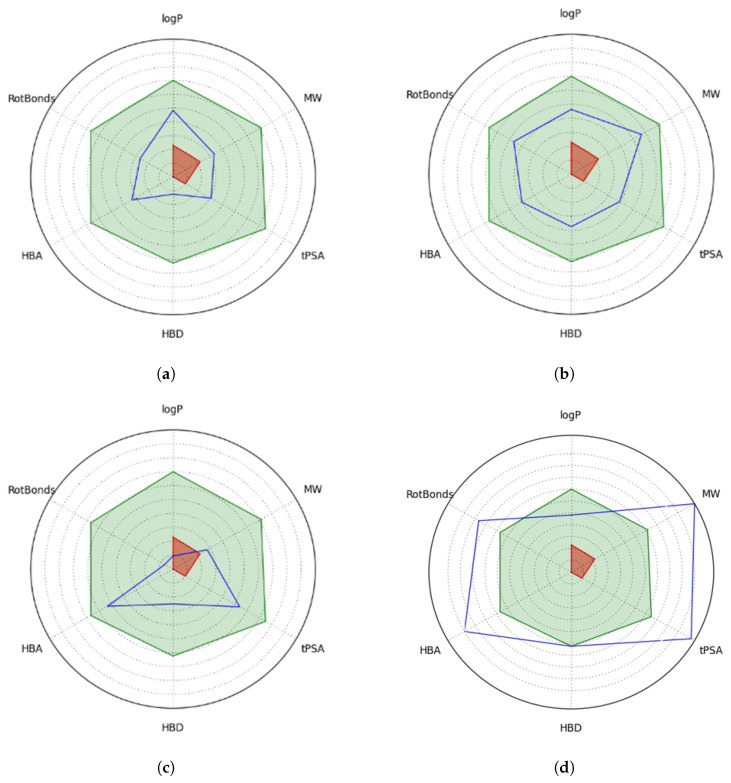
Representative radar plots of the predicted oral absorption profiles of selected approved anticancer drugs generated using FAF-Drugs4. Blue lines indicate calculated compound profiles, and green areas indicate preferred ranges for favorable oral absorption. (**a**) Lomustine and (**b**) sunitinib show profiles largely contained within the preferred region. (**c**) Temozolomide shows a deviation mainly related to low lipophilicity. (**d**) Docetaxel displays multiple deviations from the optimal oral absorption range.

Figure 4 displays representative radar plots illustrating the predicted oral absorption profiles of selected approved anticancer drugs. In each plot, the blue line denotes the calculated profile for an individual compound, while the shaded area marks the preferred range for favorable oral absorption. Compounds with calculated profiles that remain predominantly within the shaded region demonstrate greater compliance with the physicochemical criteria for oral drug-likeness. In contrast, parameters extending beyond this region indicate deviations from the optimal oral absorption profile.

Among the representative compounds, lomustine (Figure 4a) and sunitinib (Figure 4b) exhibited profiles predominantly within the optimal region, suggesting broad compatibility with favorable oral absorption characteristics. In contrast, temozolomide (Figure 4c) deviated primarily due to low lipophilicity, whereas docetaxel (Figure 4d) demonstrated several parameters outside the preferred range, particularly those associated with molecular size, polarity, and structural complexity. These results are consistent with the overall trends observed in the analyzed dataset.

Most of the anticancer drugs investigated demonstrated oral absorption profiles compatible with the preferred physicochemical space. Docetaxel and paclitaxel exhibited the most pronounced deviations, with multiple parameters outside the optimal range. Trametinib deviated primarily due to its molecular weight, while temozolomide and trifluridine displayed reduced lipophilicity compared to the optimal area. The remaining compounds were generally located within the oral absorption space, which is associated with desirable drug-like properties. Oral absorption radar plots for all investigated compounds are presented in Figure S4.

### 2.5. Toxicity Assessment

Toxicity-related positioning was evaluated using the FAF-Drugs4 toxicity space representation, which is based on lipophilicity and topological polar surface area. Within this model, compounds are distributed across regions corresponding to varying predicted toxicity levels, from lower-risk zones to areas of high toxicity.

**Figure 5:**
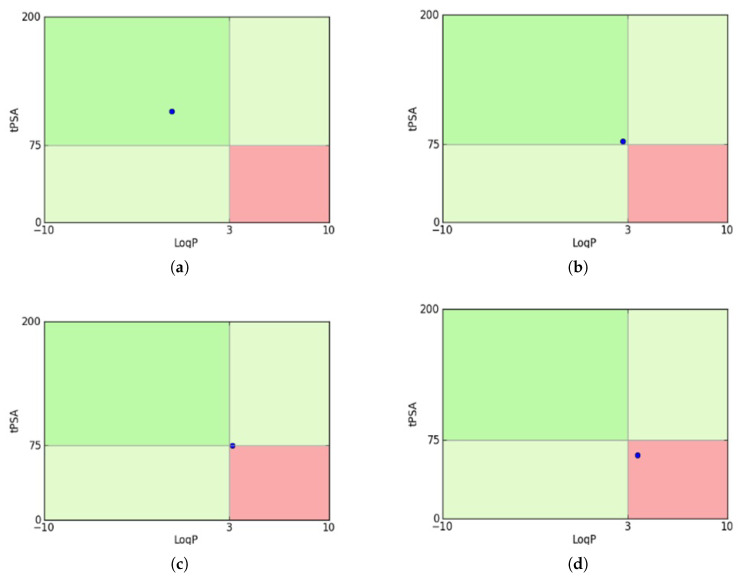
Representative toxicity space plots of selected approved anticancer drugs generated with FAF-Drugs4. Blue points indicate the analyzed compounds, green regions indicate lower-risk toxicity space areas, and the red region indicates the high-risk toxicity space area. (**a**) Temozolomide is positioned in the non-toxic region. (**b**) Sunitinib is located close to the boundary of the lower-risk space. (**c**) Erlotinib lies near the border between low- and high-toxicity regions. (**d**) Vandetanib is clearly positioned within the high-toxicity region.

Figure 5 presents representative toxicity space plots for selected approved anticancer drugs. In these plots, each compound appears as a blue point, positioned according to its predicted lipophilicity and topological polar surface area. Compounds in the dark green region are classified as non-toxic, whereas those in the light green regions are associated with lower predicted toxicity. In contrast, compounds located in the red region are predicted to present a higher toxicity risk.

Among the representative compounds, temozolomide (Figure 5a) was positioned outside the high-risk toxicity space region, indicating a favorable toxicity profile according to the FAF-Drugs4 classification. Sunitinib (Figure 5b) was situated near the boundary separating lower-risk and higher-risk regions, while erlotinib (Figure 5c) was positioned close to the border between low-toxicity and high-toxicity areas. In contrast, vandetanib (Figure 5d) was distinctly located within the high-toxicity region, representing the most significant toxicity outlier among the compounds analyzed.

Among the analyzed drugs, temozolomide, trifluridine, docetaxel, lenvatinib mesylate, and sunitinib were classified within the non-toxic region. Cabozantinib S-malate and sorafenib were identified in the low-toxicity region. Carmustine, dabrafenib, trametinib, lomustine, and erlotinib were situated near the boundaries between toxicity regions. Vandetanib was the only compound distinctly classified in the high-toxicity region. Comprehensive toxicity plots for all compounds are presented in Figure S5.

### 2.6. Predicted Pharmacokinetic Properties

We employed SwissADME to generate complementary pharmacokinetic predictions for the approved anticancer drugs under investigation. These predictions included human gastrointestinal absorption (HIA), blood–brain barrier (BBB) permeation, P-glycoprotein (P-gp) substrate status, cytochrome P450 inhibition profile, and skin permeation expressed as log $K_{p}$.

Table 1 presents the predicted pharmacokinetic properties. High gastrointestinal absorption was observed for carmustine, lomustine, trametinib, vandetanib, trifluridine, erlotinib, and sunitinib. In contrast, temozolomide, dabrafenib, docetaxel, paclitaxel, cabozantinib S-malate, lenvatinib mesylate, and sorafenib demonstrated low predicted gastrointestinal absorption. According to SwissADME, carmustine, vandetanib, erlotinib, and sunitinib are predicted to permeate the blood–brain barrier. Regarding transporter-related properties, docetaxel, paclitaxel, sunitinib, and lenvatinib mesylate are predicted to be P-gp substrates. The cytochrome P450 inhibition profile suggests that several tyrosine kinase inhibitors are associated with multiple predicted CYP interactions; notably, erlotinib and sorafenib are predicted to inhibit several CYP isoforms. Predicted skin permeation values ranged from −9.23 cm/s for docetaxel to −5.70 cm/s for vandetanib, reflecting variability in permeability-related behavior among the investigated compounds.

SwissADME BOILED-Egg plots offer a graphical method for visualizing passive gastrointestinal absorption and blood–brain barrier (BBB) permeation as functions of lipophilicity and polarity. In this model, compounds located in the white region are predicted to exhibit a higher probability of gastrointestinal absorption, while those in the yellow yolk are predicted to have a greater likelihood of BBB permeation. The color of each dot indicates P-glycoprotein (P-gp) substrate status: blue dots denote predicted P-gp substrates and red dots denote predicted P-gp non-substrates. This approach enables simultaneous interpretation of passive absorption, BBB permeation, and the likelihood of active efflux [29].

**Figure 6:**
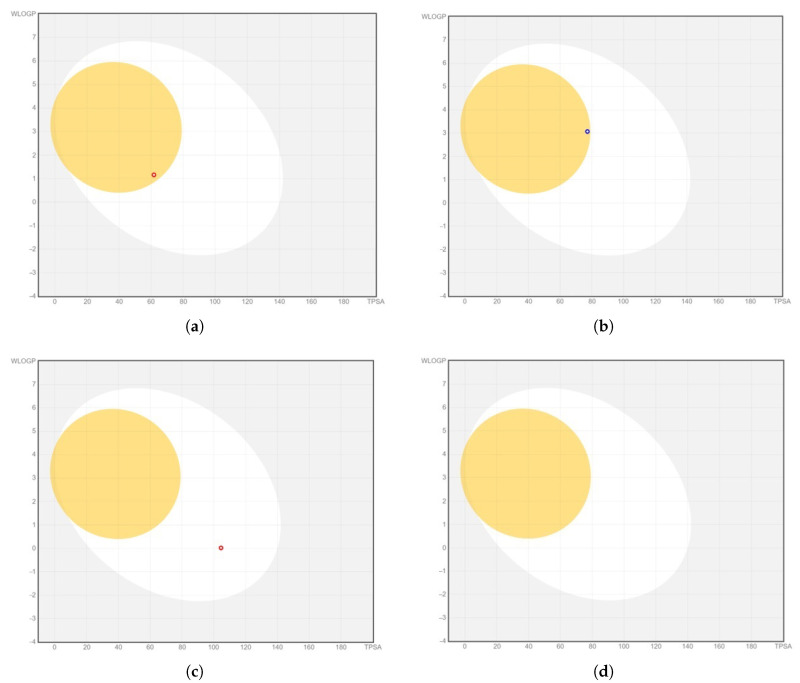
Representative BOILED-Egg plots of selected approved anticancer drugs generated with SwissADME. The white area indicates predicted gastrointestinal absorption, the yellow area indicates predicted BBB permeation, and dot color indicates P-gp substrate status. (**a**) Carmustine is positioned in the yolk as a non-P-gp substrate. (**b**) Sunitinib is positioned in the yolk as a P-gp substrate. (**c**) Trifluridine is located in the white region. (**d**) Docetaxel is positioned outside the preferred BOILED-Egg regions.

Figure 6 presents representative BOILED-Egg plots. Carmustine (Figure 6a), located in the yolk as a non-P-gp substrate, demonstrates predicted blood–brain barrier (BBB) permeation without active efflux. Sunitinib (Figure 6b) also appears in the yolk, but as a P-gp substrate, suggesting that BBB permeation could be offset by efflux. Trifluridine (Figure 6c) is situated in the white region, which aligns with high gastrointestinal absorption and a lack of predicted BBB permeation. In contrast, docetaxel (Figure 6d) is positioned outside the preferred BOILED-Egg regions and is also predicted to be a P-gp substrate, which is consistent with limited oral and central nervous system exposure.

Among the investigated compounds, carmustine, vandetanib, and erlotinib demonstrated both high gastrointestinal absorption and predicted blood–brain barrier (BBB) permeation, without P-glycoprotein (P-gp) efflux. Sunitinib also exhibited predicted BBB permeation but was identified as a P-gp substrate. Lomustine, trametinib, and trifluridine were predicted to have high gastrointestinal absorption without BBB permeation. In contrast, temozolomide, dabrafenib, cabozantinib S-malate, sorafenib, docetaxel, paclitaxel, and lenvatinib mesylate did not display the combined BOILED-Egg pattern indicative of strong gastrointestinal absorption or BBB penetration in the categorical output. Complete BOILED-Egg plots for all investigated compounds are presented in Figure S6.

### 2.7. Concordance with Regulatory and Curated Reference Data

To provide an integrated assessment of translational relevance, we compared the main in silico ADMET and pharmacokinetic patterns with regulatory and curated reference data from FDA and EMA-approved product information, as well as from DrugBank and PubChem. Table 2 summarizes the level of agreement for each drug, and the full list of underlying reference sources is provided in Table S1.

The highest concordance was identified for lomustine, vandetanib, docetaxel, and paclitaxel. Lomustine demonstrated favorable oral absorption according to FAF-Drugs4 and Golden Triangle profiles, as well as high gastrointestinal absorption in SwissADME, consistent with its capsule formulation and reported rapid gastrointestinal uptake [30,31]. Vandetanib exhibited a compatible oral profile, high gastrointestinal absorption, predicted blood–brain barrier permeation, and a strong toxicity signal, aligning with its film-coated tablet formulation, slow oral absorption, and regulatory warnings regarding QT-related cardiotoxicity [32,33]. Conversely, docetaxel and paclitaxel showed significant deviations in physicochemical and oral absorption parameters, reflecting their clinical use via infusion and limited oral suitability [34,35,36,37,38,39,40,41].

Several compounds demonstrated only partial concordance between in silico predictions and regulatory or curated data. Carmustine illustrates this, as it displays favorable FAF-Drugs4 permeability and metabolic stability, along with high gastrointestinal absorption and predicted blood–brain barrier permeation in SwissADME. However, regulatory and curated sources report an intravenous formulation and limited bioavailability [42,43,44]. Temozolomide similarly exhibited partial agreement; descriptor-based limitations related to lipophilicity and permeability, as well as low predicted gastrointestinal absorption, in contrast with regulatory and curated evidence of rapid and complete gastrointestinal absorption [45,46,47]. Trifluridine also presented a lipophilicity-related deviation despite high gastrointestinal absorption in SwissADME, a finding that aligns with its oral formulation and reported aqueous solubility [48,49,50,51].

The majority of other compounds exhibited partial concordance, suggesting that freely accessible ADMET tools are more effective at identifying broad descriptor-driven trends than at capturing clinically nuanced behaviors influenced by formulation, solubility, transporter activity, or intricate toxicity profiles.

To complement this qualitative concordance framework, selected binary endpoints were further evaluated using predefined coding rules and exploratory quantitative metrics. The main findings of this quantitative assessment are presented below, while the complete per-drug classifications, confusion matrices, performance metrics, and endpoint-specific caveats are provided in the Supplementary Materials (Tables S2–S6).

#### Exploratory Quantitative Concordance Assessment and ADME-Score

To complement the qualitative concordance assessment, selected binary endpoints were evaluated quantitatively using the predefined coding rules described in Section 4.4. Table 3 reports the confusion matrix counts and derived performance metrics for these selected endpoints. The strongest performance was for SwissADME P-gp substrate status, which showed complete agreement with the benchmark coding in this 14-drug dataset. Accuracy, sensitivity, specificity, precision, F1 score, and Cohen’s kappa were all 1.000. SwissADME gastrointestinal absorption showed moderate performance, with accuracy of 0.571, sensitivity of 0.545, specificity of 0.667, precision of 0.857, F1 score of 0.667, and Cohen’s kappa of 0.143. FAF-Drugs4 high-risk toxicity classification showed high specificity and precision, both 1.000, but low sensitivity of 0.167. This indicates that positive high-risk toxicity calls were selective but several clinically relevant toxicity liabilities were not captured. FAF-Drugs4 Golden Triangle positioning showed limited concordance with practical oral use classification, with accuracy of 0.286, sensitivity of 0.182, F1 score of 0.286, and Cohen’s kappa of −0.077.

This quantitative assessment complemented the graphical interpretation of the ADMET plots. Specifically, Golden Triangle positioning and FAF-Drugs4 toxicity space classification were treated as visual outputs converted into predefined binary categories for exploratory analysis. Thus, the figures were linked to endpoint-level concordance metrics summarized in Table 3.

SwissADME gastrointestinal absorption and FAF-Drugs4 Golden Triangle positioning both provide information on oral suitability. Their paired correctness was compared exploratorily using an exact McNemar test. SwissADME HIA correctly classified 8 out of 14 compounds, while FAF-Drugs4 Golden Triangle positioning correctly classified 4 out of 14. The discordant correctness comparison was not statistically significant, reflecting the limited power of the 14-compound benchmark. This comparison was interpreted cautiously because the two endpoints are not mechanistically identical: SwissADME HIA estimates gastrointestinal absorption, while the Golden Triangle is a medicinal chemistry heuristic related to permeability and metabolic stability.

Beyond the binary endpoint analysis, the exploratory ADME-Score provided a weighted compound-level ranking metric integrating HIA, TPSA, and LogP information. This score was not used as a formal validation endpoint but as a complementary quantitative layer to distinguish compounds with broadly favorable ADME profiles from those requiring additional interpretation due to discordant absorption, lipophilicity, or polarity features.

## 3. Discussion

This study explored the potential translational utility and limitations of two freely accessible computer-assisted ADMET platforms, SwissADME and FAF-Drugs4, by comparing their predictions with regulatory and curated reference data for 14 approved anticancer drugs. Within this focused benchmark, these platforms were mainly informative for identifying broad descriptor-driven trends related to physicochemical properties, oral suitability, permeability, metabolic stability, and general toxicity risk. Comparison with data from the FDA, EMA, DrugBank, and PubChem also delineated the limitations when clinical behavior is shaped by formulation, transport mechanisms, or complex organ-specific toxicities. Therefore, this work provides a focused case-based analysis of computer-assisted ADMET prediction in a clinically established drug set, emphasizing both its translational utility and its limitations within real-world drug development workflows.

### 3.1. Physicochemical Space and Oral Suitability

A key finding of this analysis is that the computational platforms identified compounds with physicochemical properties unfavorable for oral drug delivery. Docetaxel and paclitaxel exemplify this concordance, as both molecules exhibit significant deviations from the optimal physicochemical space, particularly regarding molecular weight, topological polar surface area, hydrogen bond acceptor count, and molecular flexibility (Figure 1). Both compounds are administered clinically as intravenous formulations [34,35]. This concordance is consistent with the potential utility of descriptor-based tools for identifying compounds with poor oral suitability at early stages of drug development [3,26].

By contrast, several orally active small molecules remained broadly compatible with the preferred physicochemical space, indicating that descriptor-based tools can also support the identification of compounds with general oral drug-like characteristics. However, the relationship between physicochemical compliance and real-world oral performance is not one-to-one and is discussed in more detail in Section 3.4.

### 3.2. Structural Complexity and Medicinal Chemistry Interpretation

Structural complexity analysis indicates that many clinically successful anticancer agents occupy a chemical space distinct from the optimal range typically associated with conventional oral drug-likeness (Figure 2 and Figure S2). For example, taxanes exhibit significant deviations in complexity-related parameters, such as high molecular weight, multiple chiral centers, and increased flexibility, yet remain clinically valuable and widely utilized. This finding underscores a broader principle in medicinal chemistry: compounds may achieve therapeutic success despite violating canonical drug-likeness filters if their pharmacological activity, formulation strategy, or route of administration compensates for unfavorable molecular properties [25,26,27].

This consideration is especially relevant in oncology, where highly potent molecules may progress through development despite suboptimal physicochemical properties if their efficacy warrants the associated complexity in administration or toxicity management. Therefore, the results should not be construed as evidence that deviations from drug-likeness criteria necessarily predict therapeutic failure. Instead, these deviations should be regarded as indicators of potential development challenges, including formulation complexity, route limitations, or the requirement for intensified safety monitoring. Accordingly, computational ADMET filtering is most valuable when used to identify potential liabilities early, not to exclude promising compounds in an absolute manner [8,26,27].

### 3.3. Interpretation of Permeability and Metabolic Stability

The Golden Triangle analysis offers a robust framework for evaluating the interplay among molecular size, lipophilicity, permeability, and clearance [28]. Carmustine, lomustine, and sunitinib are located within a region characterized by favorable permeability and metabolic stability, indicating that the computational models reflect key physicochemical properties relevant to drug exposure (Figure 3a,b and Figure S3). Conversely, dabrafenib, trametinib, vandetanib, erlotinib, cabozantinib, and sorafenib are positioned outside the optimal region, which suggests a propensity for reduced metabolic stability or less favorable clearance properties (Table 2, Figure S3). These results align with broader observations that many orally active kinase inhibitors occupy a more complex physicochemical space than classical oral drugs and frequently necessitate formulation optimization or precise clinical dosing strategies [26,27].

These findings highlight that Golden Triangle positioning is useful for identifying permeability and clearance-related trends, but should not be interpreted as a definitive representation of clinical pharmacokinetic behavior, particularly for compounds whose exposure is shaped by solubility, formulation, pH-dependent stability, or transporter-mediated processes [6,20,26,28,52].

### 3.4. Mechanistic Interpretation of Oral Absorption

The oral absorption predictions generated by FAF-Drugs4 represented some of the most informative results in our study, particularly when considered alongside regulatory and curated reference data. Overall, the computational model effectively identified compounds with physicochemical properties that are incompatible with oral administration. Notably, docetaxel and paclitaxel exhibited multiple deviations from the optimal oral absorption parameters, especially regarding molecular weight, polarity, and bond-related descriptors (Figure 4d and Figure S4). This prediction is corroborated by external reference data: both drugs are listed in EMA and FDA product information as infusion formulations [34,35,36,37], while DrugBank and PubChem report poor aqueous solubility, very high molecular weight, and elevated topological polar surface area [38,39,40,41]. Therefore, these findings demonstrate the poor oral suitability of the taxanes from both computational and clinical perspectives.

A strong concordance between computational predictions and real-world use was observed for lomustine. Its radar profile remained largely within the preferred oral absorption region, aligning with external sources (Figure 4a). The European Medicines Agency (EMA) describes lomustine as a hard capsule [30]. DrugBank reports that it is well and rapidly absorbed from the gastrointestinal tract [31]. PubChem indicates a relatively low molecular weight and moderate topological polar surface area, supporting a physicochemical profile compatible with oral therapy [53]. Lomustine exemplifies a case where computational prediction, dosage form, and curated pharmacokinetic information agree.

Trametinib provides a more nuanced case. The computational analysis identified deviation primarily due to its molecular weight (Figure S4). Reference data indicate that trametinib is marketed as an oral tablet [54]. DrugBank reports a mean absolute bioavailability of 72% for tablets and 81% for oral solution, and notes that trametinib is rapidly absorbed after oral administration [55]. PubChem confirms its relatively high molecular weight [56], while DrugBank reports limited aqueous solubility [55]. These findings indicate that the computational model correctly identifies a size-related liability, but that this liability does not preclude clinically effective oral delivery.

The most instructive mismatch was observed for temozolomide. In the FAF-Drugs4 oral absorption radar, temozolomide deviated mainly because of low lipophilicity, which may suggest less favorable passive diffusion (Figure 4c). However, EMA lists hard capsules, FDA also lists an intravenous formulation, and DrugBank states that temozolomide is rapidly and completely absorbed in the gastrointestinal tract. DrugBank additionally reports relatively high aqueous solubility, while PubChem shows a low molecular weight despite a somewhat elevated topological polar surface area. These data indicate that low lipophilicity alone does not adequately predict oral performance for this compound and that the overall absorption profile is influenced by favorable solubility and other physicochemical determinants not captured by a single descriptor. A similar, although less pronounced, limitation is seen for trifluridine. In the computational model, trifluridine also showed reduced lipophilicity relative to the preferred region (Figure S4); yet, the regulatory and curated sources indicate an oral tablet formulation, and both FDA-linked product information and DrugBank describe the compound as soluble in water (Table S1). PubChem further shows a substantially lower molecular weight than the taxanes and a more moderate size overall. In this case, the oral absorption prediction correctly identifies a lipophilicity-related deviation, but the real-world data indicate that this does not prevent oral administration.

For several orally administered kinase inhibitors, including vandetanib, erlotinib, sunitinib, cabozantinib, lenvatinib, and sorafenib, the comparison highlights a recurring pattern. These compounds are clinically developed as oral agents despite having one or more physicochemical liabilities, particularly limited aqueous solubility or relatively high molecular weight. Vandetanib, for example, is described by EMA as a film-coated tablet with slow oral absorption [32], while DrugBank reports extremely low aqueous solubility [57]. Sorafenib is also oral, although described as very poorly water-soluble and lipophilic; cabozantinib and lenvatinib similarly occupy a more challenging physicochemical space than classical orally optimized molecules (Figure S4, Table S1). These examples show that the computational model is useful for identifying oral developability challenges, but the FDA and EMA labels, as well as DrugBank and PubChem data, make it clear that such challenges can often be successfully addressed in marketed products through careful formulation strategies and clinical dose design.

The oral absorption analysis indicates that descriptor-based tools are useful for flagging oral developability challenges associated with high molecular weight, increased polarity, or poor solubility [3,15,26]. However, regulatory and curated reference data remain essential for interpreting cases in which oral administration is clinically successful despite descriptor-level deviations (Table 2 and Table S1).

The observed discrepancies require chemical class-specific mechanistic interpretation. Temozolomide shows why lipophilicity alone may fail as an absorption predictor. Despite low LogP, its low molecular weight and favorable aqueous solubility support rapid gastrointestinal absorption [47]. By contrast, taxanes have very high molecular weight, high polarity, poor solubility, and P-gp substrate status. This explains their poor oral suitability and infusion-based clinical use [38,39,40,41]. Kinase inhibitors show another class-specific pattern. Oral development may coexist with solubility, CYP, transporter, and protein binding liabilities that require formulation and dosing optimization. These examples reinforce that ADMET outputs should be interpreted in relation to chemical class, molecular mechanism, and clinical development context, not as isolated descriptors.

### 3.5. Toxicity Prediction and Clinical Safety Relevance

The toxicity-related outputs generated by FAF-Drugs4 were informative as broad early-warning signals, but their interpretation becomes much more meaningful when integrated with regulatory data and the published clinical literature. In this study, FAF-Drugs4 classified most compounds within the non-toxic or low-toxicity regions; vandetanib was the only drug clearly positioned in the high-toxicity quadrant (Figure 5d). This result is particularly relevant because vandetanib is also one of the strongest examples of concordance between in silico prediction and documented clinical risk. Both the EMA product information and the FDA label highlight the cardiotoxic potential of vandetanib, including QTc prolongation, with the FDA explicitly warning about torsades de pointes and sudden death [32,33]. This risk aligns with existing literature indicating that QT prolongation is a clinically significant adverse effect of vascular endothelial growth factor receptor tyrosine kinase inhibitors [58].

The case of erlotinib is more nuanced, but it still supports the relevance of the computational signal. In the FAF-Drugs4 plot, erlotinib was positioned near the border between the low and high-toxicity regions rather than as an extreme outlier (Figure 5c). This intermediate positioning is broadly consistent with the regulatory data: the EMA product information reports serious cases of drug-induced liver injury [59], whereas the FDA label highlights pulmonary toxicity [59]. At the same time, published clinical studies indicate that erlotinib, like other tyrosine kinase inhibitors, may also be associated with QTc prolongation. Kloth et al. reported clinically relevant QTc prolongation in patients receiving tyrosine kinase inhibitors (TKIs), including erlotinib [60], and Abu Rmilah et al. reported QTc prolongation in 24.1% of erlotinib-treated patients, compared with 80% of vandetanib-treated patients [61]. These findings suggest that erlotinib represents a clinically relevant but less pronounced cardiotoxicity signal than vandetanib, which is consistent with its borderline position in the FAF-Drugs4 toxicity space.

At the same time, our study also shows the limits of descriptor-based toxicity models. Several drugs positioned in the non-toxic or lower-risk regions still carry important warnings in regulatory labeling. Temozolomide, for example, was positioned in the non-toxic region (Figure 5a), yet the FDA label reports fatal and severe hepatotoxicity [46]. Sunitinib was likewise placed in the non-toxic region or near the lower-risk boundary (Figure 5b), although its FDA prescribing information includes a warning for hepatotoxicity [62]. Similarly, carmustine and lomustine do not emerge as the strongest toxicity outliers in the FAF-Drugs4 plot (Figure S5). However, regulatory sources describe clinically significant pulmonary toxicity for both carmustine and lomustine [30,42,43,63]. In the case of lomustine, the FDA and EMA documentation also reports risks of secondary malignancies, nephrotoxicity, embryo–fetal toxicity, and hepatotoxicity [30,63]. These discrepancies indicate that the toxicity space model is more suitable for identifying broad physicochemical liability than for predicting the full spectrum of organ-specific adverse reactions observed in clinical practice.

These findings emphasize caution when interpreting FAF-Drugs4 toxicity space outputs. While the model serves as a warning signal based on physicochemical and structural alerts, it does not fully capture the biological complexity of clinical toxicity. Adverse reactions may depend on mechanisms not captured by simple descriptor-based positioning, including metabolism-mediated toxicity, reactive intermediates, transporter-mediated exposure, mitochondrial dysfunction, immune-mediated effects, cumulative dosing, tissue-specific vulnerability, and patient-level factors [8,20]. Therefore, compounds outside the high-risk toxicity region should not be seen as clinically non-toxic. Instead, the FAF-Drugs4 toxicity output should be used as an early warning layer to help prioritize compounds for further toxicological evaluation. This interpretation aligns with the exploratory quantitative analysis, where FAF-Drugs4 high-risk toxicity classification showed high specificity but low sensitivity (Table 3). This indicates that positive high-risk classifications may be selective alerts, while the absence of such a signal does not exclude relevant toxicity.

Integrating FAF-Drugs4 toxicity outputs with EMA and FDA labeling, DrugBank annotations, and clinical studies improves the interpretation of these findings. The results suggest that FAF-Drugs4 may be useful as an early warning tool for broad toxicity-related liabilities in anticancer drugs, particularly kinase inhibitors, but regulatory and clinical evidence remain essential for defining the nature and severity of actual safety risks.

### 3.6. Pharmacokinetic Properties

In addition to the descriptor-based patterns previously discussed, SwissADME generated complementary pharmacokinetic data regarding gastrointestinal absorption, blood–brain barrier permeation, P-glycoprotein substrate status, cytochrome P450 inhibition, and skin permeation [3,6,14]. These results facilitated a more nuanced interpretation of distribution-related behavior and potential interaction liabilities [20].

A prominent pattern identified was the distinction between compounds predicted to exhibit favorable gastrointestinal absorption and those with lower oral suitability. As summarized in Table 1, carmustine, lomustine, trametinib, vandetanib, trifluridine, erlotinib, and sunitinib were predicted to have high gastrointestinal absorption, whereas temozolomide, dabrafenib, docetaxel, paclitaxel, cabozantinib S-malate, lenvatinib mesylate, and sorafenib were predicted to have low gastrointestinal absorption [3]. Docetaxel and paclitaxel also demonstrated significant deviations in oral absorption space (Figure 4d and Figure S4) and are administered clinically as infusion-based formulations [34,35,36,37]. In contrast, predictions for certain orally active kinase inhibitors indicate that lower gastrointestinal absorption scores do not necessarily preclude effective oral administration. Instead, these findings highlight developability challenges that may be addressed through formulation design and clinical dosing strategies (Table 2 and Table S1).

The blood–brain barrier (BBB) predictions provided additional insights. As summarized in Table 1 and depicted by the representative BOILED-Egg plots in Figure 6 and the complete set in Figure S6, carmustine, vandetanib, erlotinib, and sunitinib were predicted to cross the BBB, while the remaining compounds were not [3]. These findings should be considered alongside P-glycoprotein (P-gp) substrate status. Notably, sunitinib was predicted to be both BBB-permeant and a P-gp substrate, indicating that passive penetration could be offset by active efflux. In contrast, carmustine, vandetanib, and erlotinib were predicted to permeate the BBB without P-gp efflux, which supports a higher likelihood of central nervous system exposure. The BOILED-Egg representations visually reinforced these distinctions by integrating permeability-related positioning with P-gp status within a single model.

Predictions regarding P-glycoprotein (P-gp) and cytochrome P450 (CYP) interactions underscore the pharmacokinetic complexity of the targeted therapies under investigation. As shown in Table 1, docetaxel, paclitaxel, sunitinib, and lenvatinib mesylate are predicted to be P-gp substrates, indicating an increased likelihood of transporter-mediated limitations in tissue retention or absorption [3]. Several tyrosine kinase inhibitors, including vandetanib, erlotinib, and sorafenib, exhibit multiple predicted CYP interactions. In contrast, compounds such as carmustine, lomustine, trifluridine, and docetaxel are not predicted to inhibit any of the evaluated CYP isoenzymes. These results demonstrate that orally active anticancer drugs can vary significantly in their physicochemical properties, interaction liabilities, and metabolic complexity, as further supported by the reference-based concordance analysis (Table 2) and the regulatory and curated sources in Table S1.

At the same time, the SwissADME pharmacokinetic outputs revealed limitations comparable to those identified in the FAF-Drugs4 analysis. Temozolomide serves as a representative example: although the categorical SwissADME output predicts low gastrointestinal absorption and limited blood–brain barrier (BBB) permeation (Table 1), regulatory and curated reference data demonstrate rapid and extensive oral absorption following capsule administration, and an intravenous formulation is also available [45,46,47].

The interpretation of computational ADMET outputs is also influenced by several clinical confounders. The predictions generated here primarily describe the parent compound and intrinsic molecular properties, whereas observed clinical behavior may depend on formulation, route of administration, transporters beyond P-gp, active metabolites, and dosing strategies [6,20]. For example, infusion-based formulation strategies are central to the clinical use of taxanes despite their poor oral suitability profiles, while kinase inhibitors may be affected by transporter- and CYP-related interaction liabilities [3,34,35,36,37,38,39,40,41]. In addition, active metabolites and dose-adjustment strategies can modify exposure, efficacy, and toxicity in ways not captured by descriptor-based predictions. These factors further support interpreting free ADMET tools as early decision-support resources rather than as substitutes for experimental or clinical pharmacokinetic evaluation [3,6,20].

This discrepancy highlights that computational pharmacokinetic predictions are valuable for identifying general trends and developability constraints but should not be interpreted independently of formulation, route-specific development strategies, or clinically observed drug behavior [3,6,20]. This limitation is also reflected in the concordance analysis in Table 2 and the regulatory and curated sources in Table S1.

### 3.7. Potential Translational Relevance for Early Drug Development

The benchmark comparison indicates that freely accessible ADMET platforms can provide practical support in early-stage drug development, especially where rapid, cost-effective, and reproducible screening is required. Their primary contribution is the identification of broad pharmacokinetic and medicinal chemistry trends prior to resource-intensive experimental studies. This utility is particularly evident in hit-to-lead progression, lead prioritization, academic drug discovery, and the preliminary evaluation of repositioning candidates [16,17,18,19,64].

Approved anticancer drugs also constitute a valuable benchmark set for assessing the translational relevance of in silico ADMET tools, as they combine structural diversity with well-documented pharmacological behavior. In addition to de novo drug discovery, these findings may be relevant to drug repositioning, where rapid in silico ADMET assessment can facilitate the prioritization of compounds with acceptable pharmacokinetic, safety, and developability profiles before more resource-intensive follow-up [18,65]. This broader perspective aligns with previous ADMET-oriented cheminformatics studies demonstrating that in silico profiling supports early prioritization and risk assessment across diverse chemical contexts [66,67,68,69,70].

The exploratory quantitative analysis further supports a cautious interpretation of the evaluated tools (Table 3). The graphical outputs should be interpreted as visual summaries of physicochemical and pharmacokinetic space. The corresponding endpoint-level quantitative interpretation is provided by predefined binary coding and performance metrics. This distinction is important because radar plots, BOILED-Egg plots, Golden Triangle plots, and toxicity space plots help medicinal chemistry interpretation but do not constitute statistical validation. Within this small benchmark, SwissADME P-gp substrate status showed the strongest agreement, whereas SwissADME gastrointestinal absorption, FAF-Drugs4 toxicity space classification, and Golden Triangle positioning required more cautious interpretation. These results reinforce the use of free ADMET tools as early triage and uncertainty-flagging resources rather than standalone classifiers.

Similarly, the ADME-Score improves transparency by providing a predefined weighted ranking metric, but it remains descriptor-based and does not capture formulation effects, active transport, metabolites, dosing strategies, or organ-specific toxicity. It should therefore be considered a supportive tool and hypothesis-generating metric.

### 3.8. Practical Decision Framework for the Use of Free ADMET Prediction Tools

Based on the concordance patterns, quantitative findings, and observed discrepancies, we propose a practical framework for using free ADMET tools as structured decision-support resources in early drug discovery (Table 4). First, define the ADMET endpoint of interest before selecting a tool, as different tools vary in endpoint coverage and interpretation levels [3,8,14]. Second, evaluate predictions across complementary outputs and do not rely on a single descriptor, considering factors such as gastrointestinal absorption, BOILED-Egg positioning, molecular weight, lipophilicity, polarity, and P-gp substrate status [3,15,29]. Third, concordant results across tools may support compound prioritization. Discordant predictions indicate uncertainty requiring further investigation through literature, computational, or experimental methods [20,26,27,28]. Fourth, consider toxicity-related outputs as early warning indicators, not direct predictors of organ-specific clinical toxicity [20,26]. Finally, integrate ADMET predictions with the decision context, such as early triage, lead optimization, toxicity flagging, drug repositioning, or prioritization for experimental testing [16,17,18,19,64,65].

This framework emphasizes that free ADMET tools are useful when supporting transparent and reproducible decision-making. Compounds with consistently favorable profiles may be prioritized for further evaluation. Compounds with clear liabilities may be flagged for optimization or targeted testing. Compounds with mixed predictions should not be automatically excluded, especially in oncology, where clinically successful drugs may deviate from classical drug-likeness criteria [25,26,27]. In this way, the framework translates the benchmark findings into practical guidance for the cautious and transparent use of free computational ADMET tools in early drug discovery.

### 3.9. Limitations of the Study

We acknowledge several limitations: (i) the benchmark set included only 14 approved anticancer drugs, which limits statistical power and prevents broad generalization across anticancer agents or wider chemical space; (ii) our study evaluated only two freely accessible computational platforms and their conclusions, and therefore, cannot be generalized to all available ADMET tools, particularly ML-based tools with different endpoint definitions, training datasets, and applicability domains; (iii) the main analysis was descriptive and concordance-based, although an exploratory quantitative analysis was also performed for selected binary endpoints; (iv) regulatory labels differ in the level of pharmacokinetic and safety detail they provide, which may affect the apparent degree of agreement between prediction and reference data; (v) the endpoints considered in the present study were intentionally broad and clinically interpretable, meaning that more specialized mechanistic endpoints were beyond the scope of the analysis.

The exploratory quantitative analysis is also limited by the small benchmark size and by the reduction of heterogeneous regulatory and curated reference data into binary reference classes.

The study relied on FDA and EMA product information, DrugBank, and PubChem as external reference sources, but did not include formal validation against an independent experimental ADMET dataset or an additional prediction platform. Future studies should use larger datasets, ideally including 50–100 or more compounds across multiple therapeutic and chemical classes, and should incorporate additional experimental datasets and prediction platforms to provide more robust statistical and external validation of free ADMET tools.

## 4. Materials and Methods

### 4.1. Study Design

We designed this study as a retrospective benchmark analysis of computational ADMET predictions using approved anticancer drugs as reference compounds.

We selected fourteen approved anticancer drugs to ensure structural and pharmacological diversity across multiple therapeutic classes, including alkylating agents, antimetabolites, taxanes, and tyrosine kinase inhibitors. Table 5 summarizes the investigated compounds, their primary classifications, and structural information.

The benchmark focused on 14 approved anticancer compounds to enable a detailed comparison between computational predictions and established reference data. The goal was to conduct a focused translational case study instead of creating a statistically powered validation dataset. This approach facilitated analysis of prediction concordance and divergence related to administration route, formulation, physicochemical properties, transporter effects, and relevant toxicity information.

The analytical strategy consisted of generating in silico ADMET profiles using two freely accessible web-based tools and comparing the resulting outputs with pharmacokinetic and safety information extracted from regulatory and curated public sources.

### 4.2. In Silico ADMET Prediction

SwissADME and FAF-Drugs4 were selected as complementary, freely accessible platforms with transparent and interpretable outputs suitable for compound-level comparison with regulatory and curated reference data. SwissADME was used for pharmacokinetic and drug-likeness predictions, including gastrointestinal absorption, blood–brain barrier permeation, P-glycoprotein (P-gp) substrate status, CYP inhibition, skin permeation, and BOILED-Egg visualization [3]. FAF-Drugs4 was used for rule and filter-based medicinal chemistry assessment, including physicochemical profiling, oral absorption descriptors, Golden Triangle positioning, and toxicity-related structural space interpretation [8]. Broader machine learning platforms, such as ADMETlab, admetSAR, ProTox-II, and ADMET-PrInt, provide additional endpoint coverage but rely on endpoint-specific models, training datasets, and applicability domains that were beyond the focused scope of the present clinically anchored benchmark [9,10,11,12].

Computational assessments were conducted using SwissADME [3] and FAF-Drugs4 [8]. Simplified Molecular Input Line Entry System (SMILES) strings were obtained from PubChem and entered into SwissADME. SwissADME predicted pharmacokinetic properties such as gastrointestinal absorption, blood–brain barrier permeation, P-glycoprotein substrate status, cytochrome P450 inhibition, skin permeation, and Brain Or IntestinaL EstimateD permeation (BOILED-Egg).

For FAF-Drugs4, molecular structures were downloaded from PubChem as SDF files and uploaded using the predefined drug-like filtering protocol. The outputs from FAF-Drugs4 were utilized to assess physicochemical space, structural complexity, permeability, metabolic stability, oral absorption, and toxicity-related parameters.

### 4.3. Reference Data Collection

Reference data were obtained from FDA-approved prescribing information, EMA product information, DrugBank, and PubChem. FDA and EMA documents served as the primary regulatory sources for dosage form, route of administration, pharmacokinetic characteristics, warnings, precautions, and clinically relevant adverse reaction profiles. DrugBank functioned as a curated secondary source for absorption, bioavailability, solubility, lipophilicity, and selected metabolism and distribution annotations. PubChem provided standardized structural and physicochemical descriptors, such as molecular weight, hydrogen bond donor and acceptor counts, rotatable bonds, and topological polar surface area.

For each drug, the following information was extracted when available: dosage form and route of administration, oral absorption or bioavailability statements, solubility and lipophilicity data, key pharmacokinetic characteristics, and major safety liabilities identified in regulatory labeling. To ensure transparency and reproducibility, the complete list of regulatory and curated reference sources for each investigated drug is provided in Table S1, including FDA-approved prescribing information, EMA product information, DrugBank webpage, and PubChem record for each compound.

Regulatory and curated sources served as external references for contextualizing computational predictions, but did not replace formal validation against independent experimental ADMET datasets.

### 4.4. Concordance Assessment and Objective Endpoint Coding

To evaluate translational relevance, we compared the computational predictions with the extracted reference data for each drug. Concordance was categorized as agreement, partial agreement, or disagreement. An agreement was defined as a clear match between the in silico predicted trend and the known pharmacokinetic or safety behavior of the compound. Partial agreement indicated that the predicted output captured the general tendency but failed to reflect an important clinical nuance. Disagreement was assigned when the computational profile was inconsistent with the known reference behavior.

To reduce subjectivity and improve reproducibility, selected endpoints with discrete computational outputs and clinically interpretable reference anchors were converted into predefined binary classes. These endpoints included SwissADME gastrointestinal absorption, SwissADME P-gp substrate status, FAF-Drugs4 high-risk toxicity classification, and FAF-Drugs4 Golden Triangle positioning. Table 6 summarizes the binary endpoint definitions and benchmark reference-class assignment rules. These rules were applied consistently across all compounds before calculating the quantitative metrics. (Expanded endpoint definitions, primary reference anchors, and detailed caveats are provided in Table S2).

To complement binary endpoint coding, an exploratory weighted ADME-Score was calculated for each compound. Equation (1) defines the ADME-Score:

(1) $$\mathrm{ADME-Score}=\left(0.4\times {\mathrm{HIA}}_{score}\right)+\left(0.3\times {\mathrm{TPSA}}_{score}\right)+\left(0.3\times {\mathrm{LogP}}_{score}\right).$$

${\mathrm{HIA}}_{score}$ was assigned according to the SwissADME gastrointestinal absorption classification, with high absorption coded as 1 and low absorption coded as 0. ${\mathrm{TPSA}}_{score}$ and ${\mathrm{LogP}}_{score}$ were assigned according to predefined drug-likeness ranges, with favorable values coded as 1 and unfavorable values coded as 0. TPSA was considered favorable when ≤140 ${Å}^{2}$, consistent with Veber-related oral drug-likeness criteria, while LogP was considered favorable when within the accepted drug-like lipophilicity range used in the filtering framework. HIA was assigned the highest weight because oral absorption is central to early pharmacokinetic suitability, whereas TPSA and LogP were included as complementary descriptors of polarity, lipophilicity, and membrane permeability potential. The resulting score ranged from 0 to 1, with higher values indicating a more favorable predicted ADME profile. The ADME-Score was used only as an exploratory ranking metric and not as a formal validation endpoint.

The qualitative concordance categories provided integrated clinical interpretation, while binary endpoint coding and the ADME-Score were used for exploratory quantitative assessment. Due to the heterogeneity of regulatory and curated reference data, these quantitative analyses were viewed as supportive and hypothesis-generating and not as formal validation.

The per-drug binary classifications used for the exploratory quantitative analysis are provided in Table S3. For the two oral suitability outputs, SwissADME gastrointestinal absorption and FAF-Drugs4 Golden Triangle positioning, paired correctness was explored using an exact McNemar test based on the binary classifications.

### 4.5. Data Analysis

The analysis was primarily descriptive and comparative. We summarized computational outputs for each drug and interpreted them in relation to the reference profile derived from regulatory and curated sources. We discussed representative examples of concordance and divergence to highlight the practical value and limitations of freely accessible ADMET tools in early drug development.

In addition to the descriptive concordance analysis, we performed an exploratory supplementary quantitative evaluation for selected binary endpoints with discrete computational outputs and clinically interpretable reference anchors. These endpoints included SwissADME gastrointestinal absorption, SwissADME P-glycoprotein substrate status, FAF-Drugs4 high-risk toxicity classification, and FAF-Drugs4 Golden Triangle positioning. For these endpoints, confusion matrices were generated, and accuracy, sensitivity, specificity, precision, F1 score, Cohen’s kappa, and Wilson 95% confidence intervals for accuracy were calculated. Paired correctness between SwissADME gastrointestinal absorption and FAF-Drugs4 Golden Triangle positioning was compared exploratorily using an exact McNemar test. Because of the small benchmark size and the necessary simplification of heterogeneous regulatory and curated reference data into binary classes, this quantitative analysis was considered supportive and hypothesis-generating rather than definitive.

All exploratory quantitative analyses were performed in R statistical software (version 4.5.2; R Core Team, 2025) on macOS Tahoe 26.4.1. Data handling, calculation, reporting, and visualization were supported using the R packages dplyr, report, ggplot2, and gridExtra; additional package and session details are provided in the Supplementary Materials.

**Figure 7:**
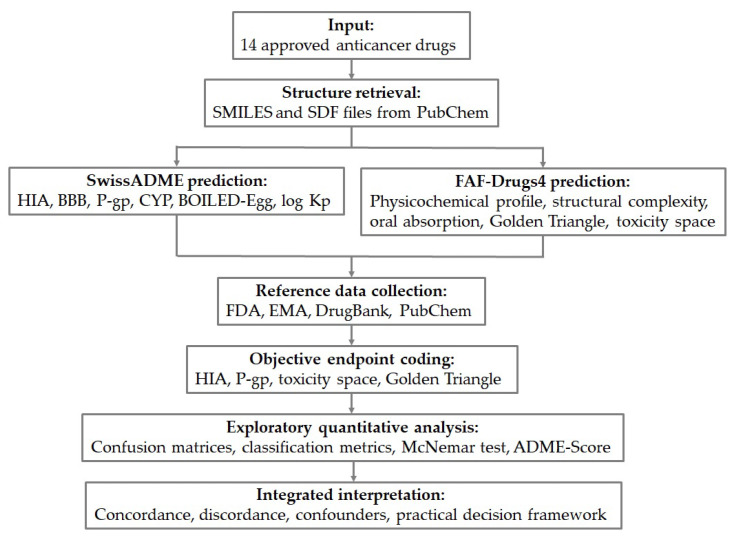
Reproducibility workflow for the computational ADMET benchmark.

The complete analytical workflow is summarized in Figure 7. The workflow includes compound selection, structure retrieval, computational prediction, reference data extraction, endpoint coding, exploratory quantitative analysis, ADME-Score calculation, and integrated interpretation.

## 5. Conclusions

This study provides exploratory evidence supporting the use of freely accessible computational ADMET platforms as effective tools for computer-assisted drug discovery. A comparison of SwissADME and FAF-Drugs4 predictions with regulatory and curated reference data for approved anticancer drugs indicates that these platforms showed the ability to identify broad descriptor-driven trends related to physicochemical space, oral suitability, permeability, metabolic stability, and general toxicity risk. The highest concordance was observed for compounds whose pharmacokinetic behavior is primarily determined by fundamental molecular properties, while lower agreement was noted for drugs whose clinical performance is influenced by formulation, transport mechanisms, or complex toxicities.

The findings support the potential use of free computational ADMET tools as efficient resources for early triage, preliminary toxicity flagging, and candidate prioritization in drug discovery workflows. These tools may also support preliminary drug repositioning by enabling rapid in silico assessment to identify approved compounds with favorable developability profiles for alternative therapeutic uses. However, these platforms should be seen as decision-support tools that complement experimental pharmacology, toxicological evaluation, and clinical evidence. This study provides a practical, case-based illustration of both the utility and limitations of computer-assisted ADMET prediction in modern drug development.

## Figures and Tables

**Table 1:** Predicted pharmacokinetic properties of the investigated approved anticancer drugs generated with SwissADME.

Drug Name	HIA	BBB	P-gp	CYP1A2	CYP2C19	CYP2C9	CYP2D6	CYP3A4	log *K_p_* (*K_p_* in cm/s)
Carmustine	High	Yes	No	No	No	No	No	No	−6.52
Lomustine	High	No	No	No	No	No	No	No	−5.72
Temozolomide	Low	No	No	No	Yes	Yes	No	Yes	−8.24
Dabrafenib	Low	No	No	No	Yes	Yes	No	Yes	−6.08
Trametinib	High	No	No	No	Yes	Yes	No	Yes	−7.62
Vandetanib	High	Yes	No	Yes	Yes	Yes	Yes	No	−5.70
Erlotinib	High	Yes	No	Yes	Yes	Yes	Yes	Yes	−6.35
Sunitinib	High	Yes	Yes	No	Yes	No	Yes	Yes	−6.86
Cabozantinib S-malate	Low	No	No	No	No	Yes	No	Yes	−7.29
Sorafenib	Low	No	No	Yes	Yes	Yes	Yes	Yes	−6.25
Lenvatinib mesylate	Low	No	Yes	No	No	Yes	No	Yes	−6.25
Docetaxel	Low	No	Yes	No	No	No	No	Yes	−9.23
Paclitaxel	Low	No	Yes	No	No	No	No	No	−8.91
Trifluridine	High	No	No	No	No	No	No	No	−8.43

Abbreviations: HIA, passive human gastrointestinal absorption; BBB, blood–brain barrier; P-gp, Permeability-glycoprotein; CYP1A2, CYP2C19, CYP2C9, CYP2D6, and CYP3A4, cytochrome P450 isoforms; $K_{p}$, skin permeability coefficient.

**Table 2:** Reference-based concordance between computational ADMET predictions and regulatory and curated data for the investigated approved anticancer drugs.

Drug Name	Main Computational Pattern	Key Reference Anchors (FDA, EMA, DrugBank, PubChem)	Overall Agreement *
Carmustine	Broadly compliant physicochemical profile; favorable permeability/metabolic-stability positioning; high GI absorption and BBB permeation; P-gp non-substrate	EMA/FDA: intravenous formulation; DrugBank/EMA: lipophilic/lipid-soluble compound with limited bioavailability; EMA: pulmonary toxicity reported	Partial agreement
Lomustine	Broadly compliant physicochemical and oral absorption profile; favorable Golden Triangle positioning; high GI absorption; no BBB permeation; no major CYP liabilities predicted	EMA: hard capsules; DrugBank: well and rapidly absorbed from the gastrointestinal tract; PubChem: low MW and moderate polarity; FDA: multiple clinically relevant toxicities	Agreement
Temozolomide	Broadly compliant oral rules, but low-lipophilicity/lower-permeability tendency; non-toxic region; low GI absorption; no BBB permeation; predicted CYP 2C19, 2C9, and 3A4 interactions	EMA/FDA: capsule formulation; FDA: also intravenous formulation; DrugBank: rapidly and completely absorbed orally; DrugBank/PubChem: low lipophilicity and low MW; FDA: severe hepatotoxicity reported	Partial agreement
Dabrafenib	Oral compatibility by general rules, but higher size/polarity burden and less favorable metabolic stability positioning; low GI absorption; no BBB permeation; predicted CYP 2C19, 2C9, and 3A4 interactions	EMA: hard capsules; DrugBank: very slightly soluble; PubChem: high MW and high polarity	Partial agreement
Trametinib	Broadly compatible oral profile with MW-related deviation; outside optimal permeability/metabolic stability region; high GI absorption; no BBB permeation; predicted CYP 2C19, 2C9, and 3A4 interactions	EMA: coated tablets; DrugBank: substantial oral bioavailability and rapid absorption; DrugBank/PubChem: low solubility and high MW	Partial agreement
Vandetanib	Broadly compatible oral profile; less favorable permeability/metabolic stability positioning; clear high-toxicity signal; high GI absorption; BBB permeation; multiple predicted CYP interactions	EMA: film-coated tablets with slow oral absorption; DrugBank: very low aqueous solubility; PubChem: relatively high MW; EMA/FDA: QT-related cardiotoxicity warnings	Agreement
Erlotinib	Broadly compatible oral profile; less favorable permeability/metabolic stability positioning; borderline toxicity signal; high GI absorption; BBB permeation; multiple predicted CYP interactions	EMA: film-coated tablets; DrugBank: very slightly soluble; EMA/FDA: liver and pulmonary toxicity warnings; clinical literature: QT-prolongation concern reported for TKIs	Partial agreement
Sunitinib	Broadly compliant physicochemical/oral profile; favorable Golden Triangle positioning; non-toxic or lower-risk positioning; high GI absorption; BBB permeation; P-gp substrate; predicted CYP 2C19, 2D6, and 3A4 interactions	EMA: hard capsules; DrugBank: lipophilic weak base with high pH-dependent solubility; FDA: hepatotoxicity warning	Partial agreement
Cabozantinib S-malate	Broadly compatible oral profile, but less favorable permeability/metabolic stability positioning and additional alert-level filters; low GI absorption; no BBB permeation; predicted CYP 2C9 and 3A4 interactions	EMA/FDA: oral formulation; DrugBank: practically insoluble in water; PubChem: high MW and high polarity	Partial agreement
Sorafenib	Broadly compatible oral profile, but less favorable metabolic stability positioning and warning-level safety signal; low GI absorption; no BBB permeation; multiple predicted CYP interactions	EMA/FDA: film-coated tablets; DrugBank: very low aqueous solubility and lipophilic distribution profile; PubChem: moderate-to-high MW	Partial agreement
Lenvatinib mesylate	Broadly compatible oral profile; borderline permeability/metabolic stability positioning; non-toxic region; low GI absorption; P-gp substrate; predicted CYP 2C9, 3A4 interactions	EMA: hard capsules; DrugBank/PubChem: relatively high MW and high polarity; DrugBank: moderate-to-high lipophilicity	Partial agreement
Docetaxel	MarkedMarked physicochemical and oral absorption deviations; major outlier for size/polarity-related properties; low GI absorption; P-gp substrate; no BBB permeation	EMA/FDA: infusion/intravenous use; DrugBank: insoluble; PubChem: very high MW and high TPSA	Agreement
Paclitaxel	Marked physicochemical and oral absorption deviations; major outlier for size/polarity-related properties; low GI absorption; P-gp substrate; no BBB permeation	EMA/FDA: infusion-based formulation; FDA/DrugBank: insoluble and highly lipophilic; PubChem: very high MW and high TPSA	Agreement
Trifluridine	Broadly compatible oral profile, but low-lipophilicity/lower-permeability tendency; non-toxic region; high GI absorption; no BBB permeation; no major CYP liabilities predicted	EMA/FDA: oral tablets; DrugBank/FDA: water-soluble compound; PubChem: moderate MW; DrugBank: low lipophilicity	Partial agreement

* Agreement was assigned descriptively based on concordance between computational predictions and regulatory and curated reference data. The complete list of underlying sources is provided in Table S1.

**Table 3:** Exploratory quantitative performance of selected binary ADMET endpoints.

Endpoint	TP	FP	FN	TN	Accuracy	Sensitivity	Specificity	Precision	F1 Score	Cohen’s κ	Wilson 95% CI
SwissADME HIA	6	1	5	2	0.571	0.545	0.667	0.857	0.667	0.143	0.326–0.786
SwissADME P-gp substrate status	4	0	0	10	1.000	1.000	1.000	1.000	1.000	1.000	0.785–1.000
FAF-Drugs4 high-risk toxicity classification	1	0	5	8	0.643	0.167	1.000	1.000	0.286	0.186	0.388–0.837
FAF-Drugs4 Golden Triangle positioning	2	1	9	2	0.286	0.182	0.667	0.667	0.286	−0.077	0.117–0.546

Abbreviations: ADMET, absorption, distribution, metabolism, excretion, and toxicity; CI, confidence interval; FAF-Drugs4, Free ADME-Tox Filtering Tool; FN, false negative; FP, false positive; HIA, human gastrointestinal absorption; P-gp, P-glycoprotein; TN, true negative; TP, true positive.

**Table 4:** Practical decision framework for the use of free ADMET prediction tools in early drug discovery.

Decision Step	Practical Question	Recommended Interpretation
Endpoint selection	Which ADMET endpoint is relevant to the research question?	Select tools according to endpoint coverage; avoid interpreting unavailable endpoints indirectly.
Physicochemical screening	Does the compound fall within drug-like descriptor space?	Use as an early developability flag, not as an exclusion rule.
Absorption/permeability assessment	Are HIA, BOILED-Egg, Golden Triangle, and oral absorption outputs concordant?	Concordant favorable predictions support prioritization; discordance indicates uncertainty.
Transporter/metabolism check	Are P-gp or CYP liabilities predicted?	Treat as interaction or exposure risk signals requiring follow-up.
Toxicity flagging	Does the compound fall into a high-risk toxicity region or show alerts?	Use as early warning only; do not infer organ-specific clinical toxicity from descriptor-based outputs.
Reference integration	Are predictions consistent with known regulatory/curated data or related compounds?	Use external data to contextualize predictions and identify clinically relevant exceptions.
Decision output	What action should the researcher take?	Prioritize, deprioritize, flag for experimental validation, or interpret as uncertain.

Abbreviations: ADMET, absorption, distribution, metabolism, excretion, and toxicity; BOILED-Egg, Brain Or IntestinaL EstimateD permeation; CYP, cytochrome P450; HIA, human gastrointestinal absorption; P-gp, P-glycoprotein.

**Table 5:** Therapeutic categories, chemical classes, and molecular formulas of the investigated approved anticancer drugs.

Drug Name	Therapeutic Category ^1^	Chemical Class ^2^	Molecular Formula ^3^
Carmustine	Alkylating drug	Nitrosoureas	$C_{5}H_{9}{\mathrm{Cl}}_{2}N_{3}O_{2}$
Lomustine	Alkylating drug	Nitrosoureas	$C_{9}H_{16}{\mathrm{ClN}}_{3}O_{2}$
Temozolomide	Alkylating drug	Imidazotetrazines	$C_{6}H_{6}N_{6}O_{2}$
Dabrafenib	Protein kinase inhibitor	Sulfanilides	$C_{23}H_{20}F_{3}N_{5}O_{2}S_{2}$
Trametinib	Protein kinase inhibitor	Pyridopyrimidines	$C_{26}H_{23}{\mathrm{FIN}}_{5}O_{4}$
Vandetanib	Protein kinase inhibitor	Quinazolinamines	$C_{22}H_{24}{\mathrm{BrFN}}_{4}O_{2}$
Erlotinib	Protein kinase inhibitor	Quinazolinamines	$C_{22}H_{23}N_{3}O_{4}$
Sunitinib	Protein kinase inhibitor	Indolines	$C_{22}H_{27}{\mathrm{FN}}_{4}O_{2}$
Cabozantinib S-malate	Protein kinase inhibitor	Diarylethers	$C_{32}H_{30}{\mathrm{FN}}_{3}O_{10}$
Sorafenib	Protein kinase inhibitor	Diarylethers	$C_{21}H_{16}{\mathrm{ClF}}_{3}N_{4}O_{3}$
Lenvatinib mesylate	Protein kinase inhibitor	Quinoline carboxamides	$C_{22}H_{23}{\mathrm{ClN}}_{4}O_{7}S$
Docetaxel	Microtubule inhibitor	Taxanes	$C_{43}H_{53}{\mathrm{NO}}_{14}$
Paclitaxel	Microtubule inhibitor	Taxanes	$C_{47}H_{51}{\mathrm{NO}}_{14}$
Trifluridine	Nucleoside metabolic inhibitor	Pyrimidine nucleosides	$C_{10}H_{11}F_{3}N_{2}O_{5}$

^1^ Therapeutic categories were assigned according to DrugBank. ^2^ Chemical class corresponds to the Direct Parent in DrugBank Chemical Taxonomy. ^3^ Molecular formulas were retrieved from PubChem.

**Table 6:** Objective endpoint coding rules used for exploratory quantitative concordance assessment.

Endpoint	Computational Coding	Benchmark Reference Coding	Interpretation Caveat
SwissADME HIA	High gastrointestinal absorption coded as positive; low gastrointestinal absorption coded as negative.	Clinically oral drugs or compounds described as meaningfully absorbed after oral administration coded as reference positive; infusion-based/non-oral agents coded as reference negative.	Reflects practical oral suitability, not direct intestinal permeability.
SwissADME P-gp substrate status	Predicted P-gp substrate coded as positive; predicted non-substrate coded as negative.	Compounds described in curated/reference sources as clinically relevant P-gp substrates coded as reference positive; others coded as reference negative.	Small sample size limits generalizability.
FAF-Drugs4 high-risk toxicity classification	Compounds located in the high-risk toxicity region coded as positive; compounds outside this region coded as negative.	Compounds with clinically important high-priority toxicity liabilities documented in regulatory/reference sources coded as reference positive.	Exploratory analogical benchmark; clinical toxicity is organ- and mechanism-specific.
FAF-Drugs4 Golden Triangle positioning	Compounds inside the Golden Triangle coded as positive; compounds outside the triangle coded as negative.	Clinically successful oral agents coded as reference positive; non-oral or infusion-based agents coded as reference negative.	Heuristic medicinal chemistry filter, not a direct clinical endpoint.

Abbreviations: FAF-Drugs4, Free ADME-Tox Filtering Tool; HIA, human gastrointestinal absorption; P-gp, P-glycoprotein.

## Data Availability

The original contributions presented in this study are included in the article and Supplementary Materials. Further inquiries can be directed to the corresponding author.

## Supplementary Materials

The following supporting information can be downloaded at: https://www.mdpi.com/article/10.3390/ph19060840/s1.

## Abbreviations

The following abbreviations are used in this manuscript:

ADMET	Absorption, distribution, metabolism, excretion, and toxicity
BBB	Blood–brain barrier
BOILED-Egg	Brain Or IntestinaL EstimateD permeation
CYP	Cytochrome P450
EMA	European Medicines Agency
FDA	U.S. Food and Drug Administration
HIA	Human gastrointestinal absorption
ML	Machine learning
MW	Molecular weight
P-gp	P-glycoprotein
QSAR	Quantitative structure–activity relationship
SMILES	Simplified Molecular Input Line Entry System
TKI	Tyrosine kinase inhibitor
tPSA	topological polar surface area
