# Supplementary material for: The Complexity of Drug Development: Translational Value and Limitations of Computational ADMET Assays Applied to Approved Anticancer Drugs
Source: Pharmaceuticals (Basel). 2026 May 28;19(6):840. doi: 10.3390/ph19060840 (PMC13305401; doi:10.3390/ph19060840)
Supplement: Supplementary file 1 [file pharmaceuticals-19-00840-s001.zip › pharmaceuticals-4315092-supplementary.pdf]

Supplementary Materials

The Complexity of Drug Development: Translational Value and Limitations of Computational ADMET Assays Applied to Approved Anticancer Drugs

Mirela Nicolov, Adina Octavia Dușe, Elena-Daniela Jurj, Daiana Colibășanu, Adrian Voicu, Claudia Watz, Mirela Voicu, and Lucreția Udrescu

Contents

Table S1. Regulatory and curated reference sources used for each investigated approved anticancer drug.
Supplementary Note S1. Exploratory quantitative evaluation of selected binary ADMET endpoints.
Supplementary Note S2. R statistical environment and software reproducibility details.
Table S2. Binary endpoint definitions and benchmark reference-class assignment rules used for the exploratory quantitative analysis.
Table S3. Per-drug binary classifications used for the exploratory quantitative analysis of selected SwissADME and FAF-Drugs4 endpoints.
Table S4. Confusion matrices for the exploratory quantitative analysis of selected binary ADMET endpoints.
Table S5. Performance metrics for the exploratory quantitative analysis, including accuracy, sensitivity, specificity, precision, F1 score, Cohen's kappa, and Wilson 95% confidence intervals for accuracy.
Table S6. Endpoint-specific interpretation notes and caveats for the exploratory quantitative analysis.
Figure S1. Radar plots of physicochemical properties for all investigated approved anticancer drugs generated with FAF-Drugs4.
Figure S2. Radar plots of structural complexity for all investigated approved anticancer drugs generated with FAF-Drugs4.
Figure S3. Golden Triangle plots for permeability and metabolic stability for all investigated approved anticancer drugs generated with FAF-Drugs4.
Figure S4. Radar plots of predicted oral absorption for all investigated approved anticancer drugs generated with FAF-Drugs4.
Figure S5. Toxicity-space plots for all investigated approved anticancer drugs generated with FAF-Drugs4.
Figure S6. BOILED-Egg plots of predicted pharmacokinetic properties for all investigated approved anticancer drugs generated with SwissADME

Table S1. Regulatory and curated reference sources used for each investigated approved anticancer drug, including FDA-approved prescribing information, EMA product information, DrugBank webpages, and PubChem records. (All accessed on 2025-09-04.)

Drug	EMA	FDA	DrugBank	PubChem
**Carmustine**	https://www.ema.europa.eu/en/medicines/human/EPAR/carmustine-medac	https://www.accessdata.fda.gov/drugsatfda_docs/label/2007/017422s037lbl.pdf	https://go.drugbank.com/drugs/DB00262	https://pubchem.ncbi.nlm.nih.gov/compound/2578#section=Chemical-and-Physical-Properties
**Lomustine**	https://www.medac.eu/fileadmin/user_upload/medac-eu/SPCs/common_SPCs/Lomustine_medac-spc-common.pdf	https://www.accessdata.fda.gov/drugsatfda_docs/label/2016/017588s042lbl.pdf	https://go.drugbank.com/drugs/DB01206	https://pubchem.ncbi.nlm.nih.gov/compound/3950#section=Chemical-and-Physical-Properties
**Temozolomide**	https://www.ema.europa.eu/en/documents/product-information/temodal-epar-product-information_en.pdf	https://www.accessdata.fda.gov/drugsatfda_docs/label/2016/021029s031lbl.pdf	https://go.drugbank.com/drugs/DB00853	https://pubchem.ncbi.nlm.nih.gov/compound/5394#section=Chemical-and-Physical-Properties
**Dabrafenib**	https://www.ema.europa.eu/en/documents/product-information/tafinlar-epar-product-information_en.pdf	https://www.accessdata.fda.gov/drugsatfda_docs/label/2022/202806s022lbl.pdf	https://go.drugbank.com/drugs/DB08912	https://pubchem.ncbi.nlm.nih.gov/compound/44462760#section=Chemical-and-Physical-Properties
**Trametinib**	https://www.ema.europa.eu/en/documents/product-information/mekinist-epar-product-information_en.pdf	https://www.accessdata.fda.gov/drugsatfda_docs/label/2022/204114s024lbl.pdf	https://go.drugbank.com/drugs/DB08911	https://pubchem.ncbi.nlm.nih.gov/compound/11707110#section=Chemical-and-Physical-Properties
**Vandetanib**	https://www.ema.europa.eu/en/documents/product-information/caprelsa-epar-product-information_en.pdf	https://www.accessdata.fda.gov/drugsatfda_docs/label/2022/022405s019lbl.pdf	https://go.drugbank.com/drugs/DB05294	https://pubchem.ncbi.nlm.nih.gov/compound/3081361#section=Chemical-and-Physical-Properties
**Docetaxel**	https://www.ema.europa.eu/en/documents/product-information/taxotere-epar-product-information_en.pdf	https://www.accessdata.fda.gov/drugsatfda_docs/label/2022/215813s000lbl.pdf	https://go.drugbank.com/drugs/DB01248	https://pubchem.ncbi.nlm.nih.gov/compound/148124#section=Chemical-and-Physical-Properties
**Trifluridine**	https://www.ema.europa.eu/en/documents/product-information/lonsurf-epar-product-information_en.pdf	https://www.accessdata.fda.gov/drugsatfda_docs/label/2023/207981s012lbl.pdf	https://go.drugbank.com/drugs/DB00432	https://pubchem.ncbi.nlm.nih.gov/compound/6256#section=Computed-Properties

**Paclitaxel**	https://www.ema.europa.eu/en/documents/product-information/abraxane-epar-product-information_en.pdf	https://www.accessdata.fda.gov/drugsatfda_docs/label/2011/020262s049lbl.pdf	https://go.drugbank.com/drugs/DB01229	https://pubchem.ncbi.nlm.nih.gov/compound/36314#section=Chemical-and-Physical-Properties
**Erlotinib**	https://www.ema.europa.eu/en/documents/product-information/tarceva-epar-product-information_en.pdf	https://www.accessdata.fda.gov/drugsatfda_docs/label/2010/021743s14s16lbl.pdf	https://go.drugbank.com/drugs/DB00530	https://pubchem.ncbi.nlm.nih.gov/compound/176870#section=Chemical-and-Physical-Properties
**Sunitinib**	https://www.ema.europa.eu/en/documents/product-information/sutent-epar-product-information_en.pdf	https://www.accessdata.fda.gov/drugsatfda_docs/label/2011/021938s13s17s18lbl.pdf	https://go.drugbank.com/drugs/DB00530	https://pubchem.ncbi.nlm.nih.gov/compound/5329102#section=Chemical-and-Physical-Properties
**Cabozantinib S-malate**	https://www.ema.europa.eu/en/documents/product-information/cabometyx-epar-product-information_en.pdf	https://www.accessdata.fda.gov/drugsatfda_docs/psg/PSG_208692.pdf	https://go.drugbank.com/drugs/DB08875	https://pubchem.ncbi.nlm.nih.gov/compound/25102846#section=Chemical-and-Physical-Properties
**Lenvatinib mesylate**	https://www.ema.europa.eu/en/documents/product-information/lenvima-epar-product-information_en.pdf	https://www.accessdata.fda.gov/drugsatfda_docs/label/2021/206947s021lbl.pdf	https://go.drugbank.com/drugs/DB09078	https://pubchem.ncbi.nlm.nih.gov/compound/11237762#section=Chemical-and-Physical-Properties
**Sorafenib**	https://www.ema.europa.eu/en/documents/product-information/nexavar-epar-product-information_en.pdf	https://www.accessdata.fda.gov/drugsatfda_docs/label/2018/021923s020lbl.pdf	https://go.drugbank.com/drugs/DB00398	https://pubchem.ncbi.nlm.nih.gov/compound/216239#section=Chemical-and-Physical-Properties

Supplementary Note S1. Exploratory quantitative evaluation of selected binary ADMET endpoints

This supplementary note provides an exploratory quantitative analysis designed to complement the descriptive concordance framework used in the main manuscript. The primary study was structured as a retrospective benchmark comparison of SwissADME and FAF-Drugs4 predictions against regulatory and curated reference data for 14 approved anticancer drugs, with interpretation focused on broad and clinically meaningful endpoints rather than formal classifier validation.

For the present supplementary analysis, four binary endpoints were selected because both the computational outputs and the benchmark reference data could be reasonably simplified into discrete classes: SwissADME gastrointestinal absorption, SwissADME P-glycoprotein substrate status, FAF-Drugs4 high-risk toxicity classification, and FAF-Drugs4 Golden Triangle positioning. Performance was explored using confusion matrices, accuracy, sensitivity, specificity, precision, F1 score, Cohen's kappa, and Wilson 95% confidence intervals for accuracy.

This supplementary analysis should be interpreted cautiously. The benchmark set is small, and several regulatory and curated reference statements required simplification into binary categories. In addition, some evaluated outputs, particularly FAF-Drugs4 toxicity space and Golden Triangle positioning, are heuristic physicochemical indicators rather than direct clinical truth labels. Accordingly, the results presented here are supportive and hypothesis-generating, and are intended to refine the interpretation of the main manuscript rather than replace the descriptive concordance analysis.

Supplementary Note S2. R statistical environment and software reproducibility details

The exploratory quantitative analyses were conducted using R statistical software (version 4.5.2; R Core Team, 2025) on macOS Tahoe 26.4.1. The analyses included construction of confusion matrices, calculation of accuracy, sensitivity, specificity, precision, F1 score, Cohen's kappa, Wilson 95% confidence intervals for accuracy, and the exploratory exact McNemar test for paired correctness comparison between SwissADME gastrointestinal absorption and FAF-Drugs4 Golden Triangle positioning.

The R computational environment included the following packages: gridExtra version 2.3, Matrix version 1.7.5, glmnet version 5.0, Biobase version 2.70.0, BiocGenerics version 0.56.0, rcellminer version 2.32.0, rcellminerData version 2.32.0, report version 0.6.4, ggplot2 version 4.0.3, dplyr version 1.2.1, and generics version 0.1.4. These details are provided to support reproducibility of the exploratory quantitative workflow.

The per-drug binary classifications used as input for the quantitative analysis are provided in Table S3. The confusion matrices are provided in Table S4, and the corresponding performance metrics are provided in Table S5. Endpoint-specific interpretation caveats are summarized in Table S6.

Table S2. Expanded endpoint definitions, benchmark reference-class assignment rules, primary reference anchors, and caveats used for the exploratory quantitative analysis.

Computational negative class	Benchmark reference positive class	Benchmark reference negative class	Primary reference anchors	Main caveat
Low gastrointestinal absorption	Compound clinically used as an oral agent and/or explicitly described in regulatory or curated sources as rapidly, extensively, or meaningfully absorbed after oral administration	Compound used primarily as infusion/intravenous therapy and not considered orally suitable in the benchmark coding	FDA/EMA dosage form and route; DrugBank oral absorption statements; Table S1	This benchmark class reflects practical oral suitability, not a direct experimental intestinal permeability value.
Predicted non-substrate	Compound classified in the benchmark coding as a clinically relevant or curated P-gp substrate	Compound not classified as a P-gp substrate in the benchmark coding	DrugBank and literature-backed coding used in the working quantitative note; Table S1	Binary coding is acceptable here, but the small sample size limits generalizability.
Compound outside the high-risk red zone	Compound assigned in the benchmark coding to a clinically important high-priority toxicity-liability class	Compound not assigned to the high-priority toxicity-liability class in the benchmark coding	FDA/EMA warnings; Table 3; Table S1	This is an exploratory analogical benchmark, because the FAF-Drugs4 toxicity-space model is physicochemical, whereas real toxicities are organ- and mechanism-specific.
Compound outside the Golden Triangle	Compound considered a clinically successful oral agent in the benchmark coding	Compound considered non-oral/infusion-based in the benchmark coding	FDA/EMA dosage form and route; DrugBank oral suitability information; Table S1	This is a heuristic medicinal-chemistry filter, not a direct clinical endpoint.

Benchmark reference classes were simplified from heterogeneous regulatory and curated evidence and were used only for exploratory supplementary analysis. They should not be interpreted as formal external validation labels.

Table S3. Per-drug binary classifications used for the exploratory quantitative analysis of selected SwissADME and FAF-Drugs4 endpoints.

Drug	HIA prediction	HIA benchmark reference	P-gp prediction	P-gp benchmark reference	FAF-Drugs4 high-risk toxicity prediction	High-priority toxicity benchmark reference	Golden Triangle prediction	Golden Triangle benchmark reference
Carmustine	Positive	Negative	Negative	Negative	Negative	Positive	Positive	Negative
Lomustine	Positive	Positive	Negative	Negative	Negative	Positive	Positive	Positive
Temozolomide	Negative	Positive	Negative	Negative	Negative	Positive	Negative	Positive
Dabrafenib	Negative	Positive	Negative	Negative	Negative	Negative	Negative	Positive
Trametinib	Positive	Positive	Negative	Negative	Negative	Negative	Negative	Positive
Vandetanib	Positive	Positive	Negative	Negative	Positive	Positive	Negative	Positive
Erlotinib	Positive	Positive	Negative	Negative	Negative	Positive	Negative	Positive
Sunitinib	Positive	Positive	Positive	Positive	Negative	Positive	Positive	Positive
Cabozantinib S-malate	Negative	Positive	Negative	Negative	Negative	Negative	Negative	Positive
Sorafenib	Negative	Positive	Negative	Negative	Negative	Negative	Negative	Positive
Lenvatinib mesylate	Negative	Positive	Positive	Positive	Negative	Negative	Negative	Positive
Docetaxel	Negative	Negative	Positive	Positive	Negative	Negative	Negative	Negative
Paclitaxel	Negative	Negative	Positive	Positive	Negative	Negative	Negative	Negative
Trifluridine	Positive	Positive	Negative	Negative	Negative	Negative	Negative	Positive

Positive/negative labels refer to the exploratory benchmark coding scheme described in Table S2. HIA and P-gp predictions were derived from SwissADME categorical outputs reported in Table 2 of the main manuscript, whereas Golden Triangle and toxicity classifications were derived from FAF-Drugs4-based analyses reported in Sections 3.3 and 3.5.

Table S4. Confusion matrices for the exploratory quantitative analysis of selected binary ADMET endpoints.

**Table S4A. SwissADME gastrointestinal absorption (HIA)**		
	Reference positive	Reference negative
Prediction positive	6	1
Prediction negative	5	2
**Table S4B. SwissADME P-glycoprotein substrate status**		
	Reference positive	Reference negative
Prediction positive	4	0
Prediction negative	0	10
**Table S4C. FAF-Drugs4 high-risk toxicity classification**		
	Reference positive	Reference negative
Prediction positive	1	0
Prediction negative	5	8
**Table S4D. FAF-Drugs4 Golden Triangle positioning**		
	Reference positive	Reference negative
Prediction positive	2	1
Prediction negative	9	2

Confusion matrices were generated from the binary classifications shown in Table S3. *Positive* and *Negative* are endpoint-specific and follow the benchmark rules summarized in Table S2.

Table S5. Performance metrics for the exploratory quantitative analysis, including accuracy, sensitivity, specificity, precision, F1 score, Cohen's kappa, and Wilson 95% confidence intervals for accuracy.

Endpoint	Accuracy	Sensitivity	Specificity	Precision	F1 score	Cohen's kappa	Wilson 95% CI for accuracy
SwissADME HIA	0.571	0.545	0.667	0.857	0.667	0.143	0.326–0.786
SwissADME P-gp substrate status	1	1	1	1	1	1	0.785–1.000
FAF-Drugs4 high-risk toxicity classification	0.643	0.167	1	1	0.286	0.186	0.388–0.837
FAF-Drugs4 Golden Triangle positioning	0.286	0.182	0.667	0.667	0.286	−0.077	0.117–0.546

Metrics were calculated from the confusion matrices shown in Table S4. Accuracy confidence intervals were estimated using the Wilson method. Because of the small benchmark size and the simplification of regulatory/curated evidence into binary classes, these metrics should be interpreted as exploratory and supportive.

Table S6. Endpoint-specific interpretation notes and caveats for the exploratory quantitative analysis.

Endpoint	Main strength observed in this benchmark	Main limitation observed in this benchmark	Practical interpretation for the manuscript
SwissADME HIA	High precision: when the model predicts high GI absorption, the prediction is often compatible with practical oral use in this benchmark.	Moderate sensitivity: several clinically oral drugs were predicted as low absorption.	Useful for supporting oral compatibility, but not reliable enough to exclude compounds from oral consideration.
SwissADME P-gp substrate status	Excellent agreement in this 14-drug set.	Very small benchmark and no obvious borderline cases.	Promising within this dataset, but should be described cautiously as benchmark-specific rather than universally validated.
FAF-Drugs4 high-risk toxicity classification	Very high specificity: the model produced no false high-risk calls in this benchmark.	Very low sensitivity: many clinically important toxic drugs were not classified in the high-risk zone.	Best interpreted as a broad physicochemical warning signal, not as a predictor of organ-specific or mechanism-specific clinical toxicity.
FAF-Drugs4 Golden Triangle positioning	High selectivity for classical orally favorable profiles.	Very low sensitivity for modern orally active anticancer agents, especially kinase inhibitors.	Best interpreted as a conservative medicinal-chemistry heuristic rather than a clinical oral-use classifier.

Figure S1. Radar plots of the physicochemical properties of the investigated approved anticancer drugs generated with FAF-Drugs4. Each panel corresponds to one investigated drug. The dark blue line represents the calculated physicochemical profile of the compound, whereas the light blue shaded area indicates the optimal physicochemical space generally associated with drug-like molecules.

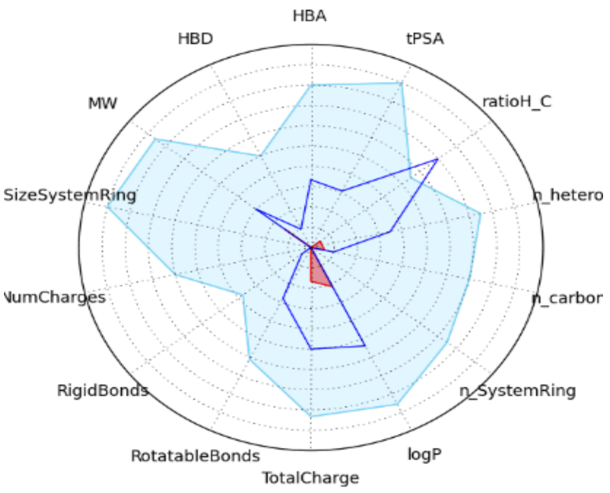

Carmustine

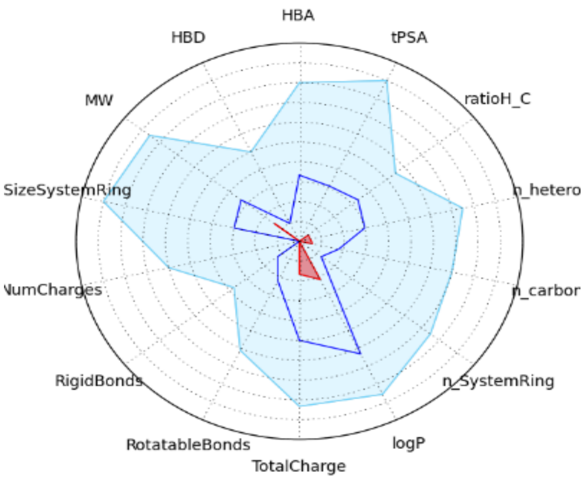

Lomustine

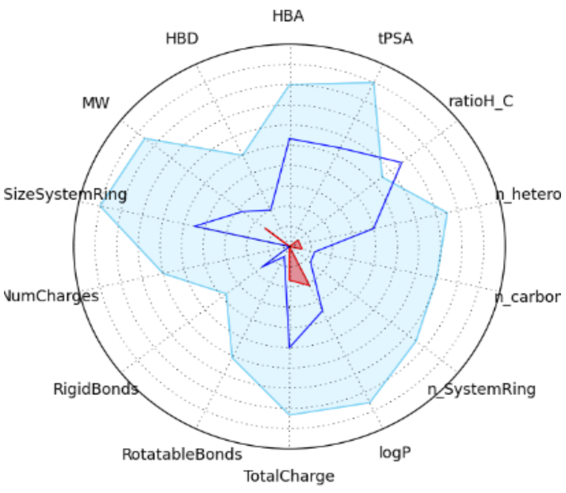

Temozolomide

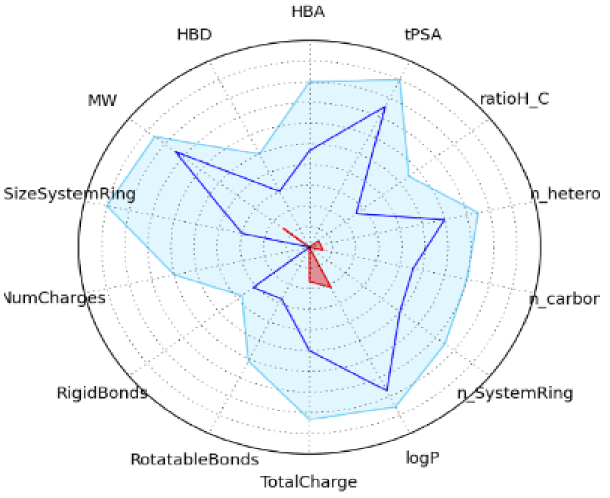

Dabrafenib

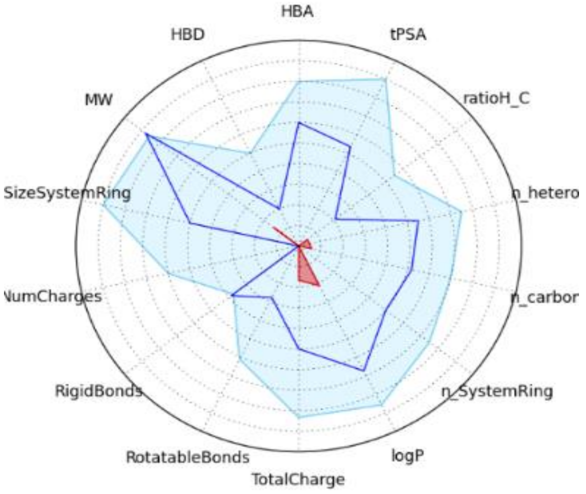

Trametinib

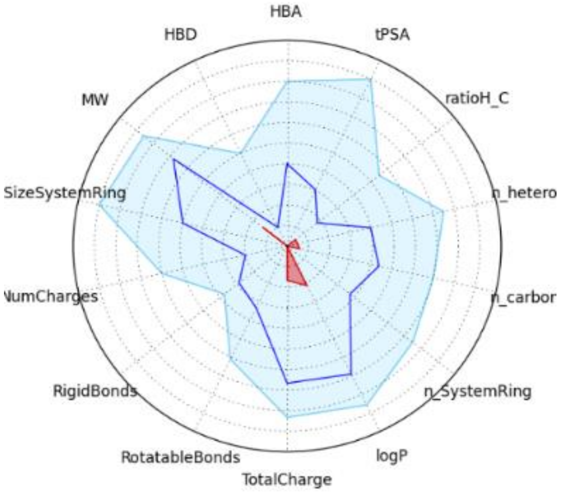

Vandetanib

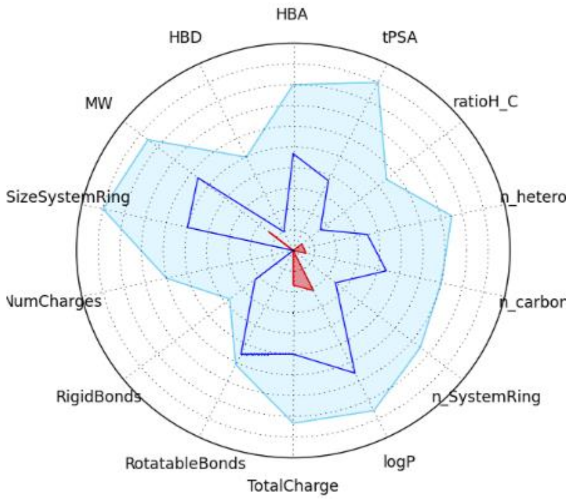

Erlotinib

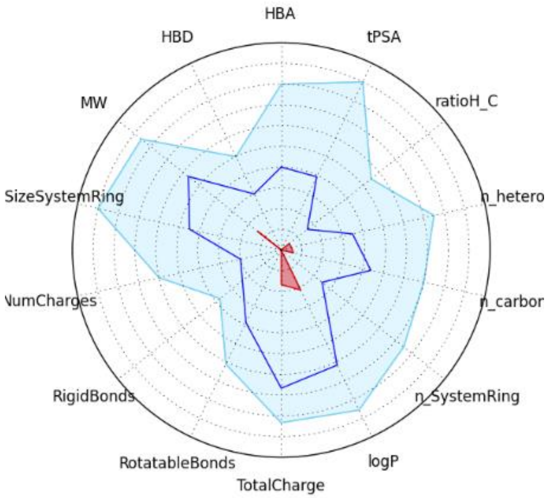

Sunitinib

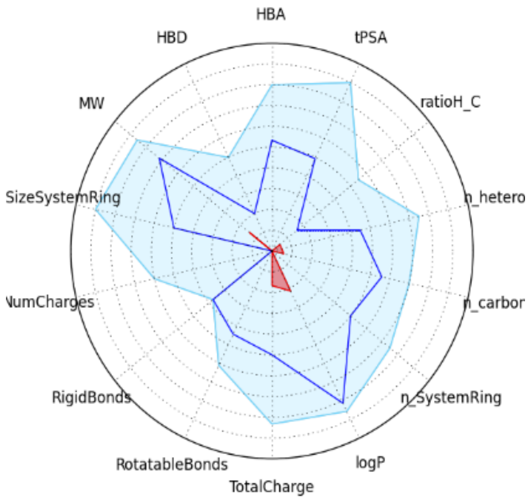

Cabozantinib S-malate

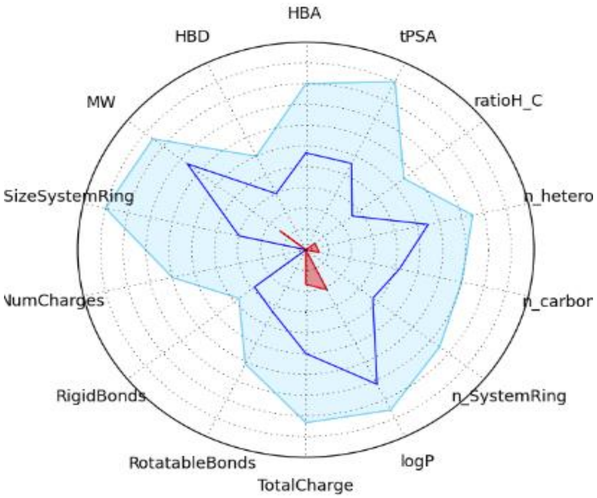

Sorafenib

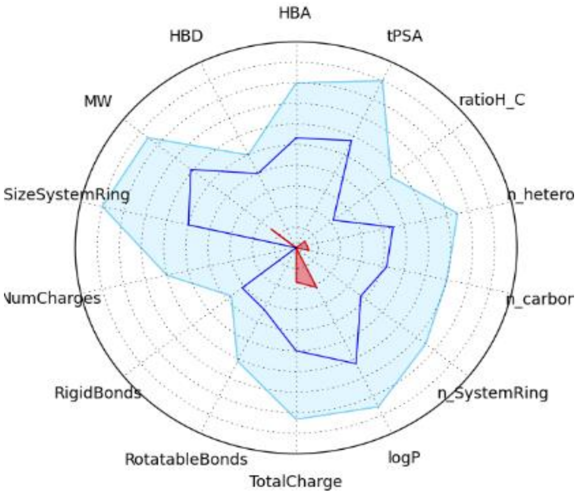

Lenvatinib mesylate

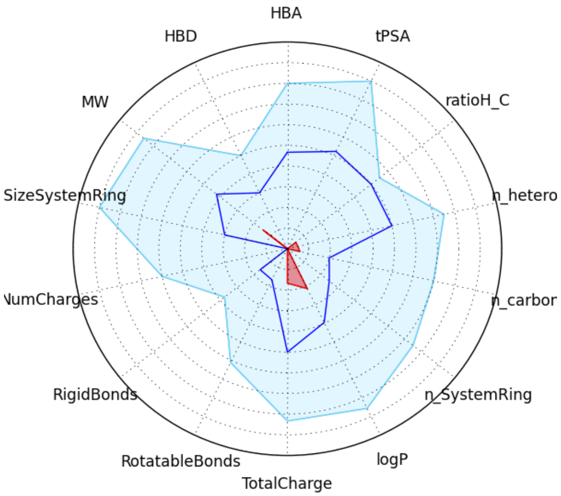

Trifluridine

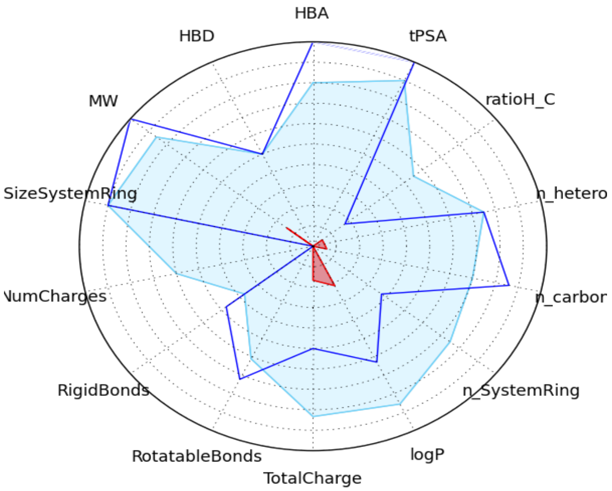

Docetaxel

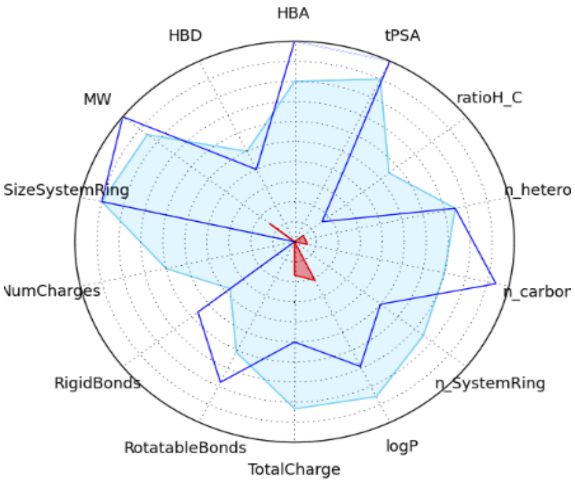

Paclitaxel

Figure S2. Radar plots of the structural complexity profiles of the investigated approved anticancer drugs generated with FAF-Drugs4. Each panel corresponds to one investigated drug. The blue line represents the calculated structural complexity profile of the compound, whereas the light-red shaded area indicates the preferred range for drug-like molecules.

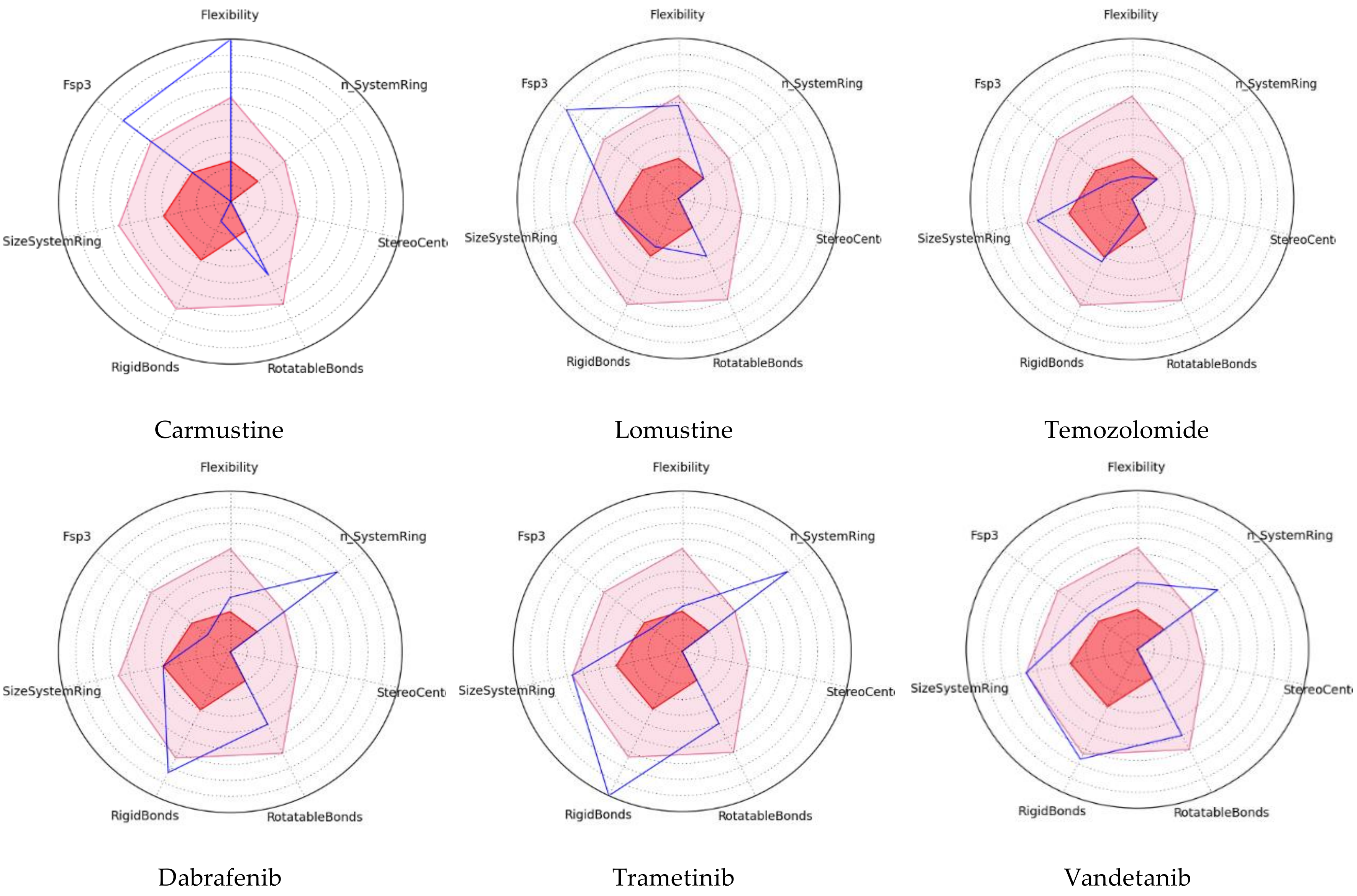

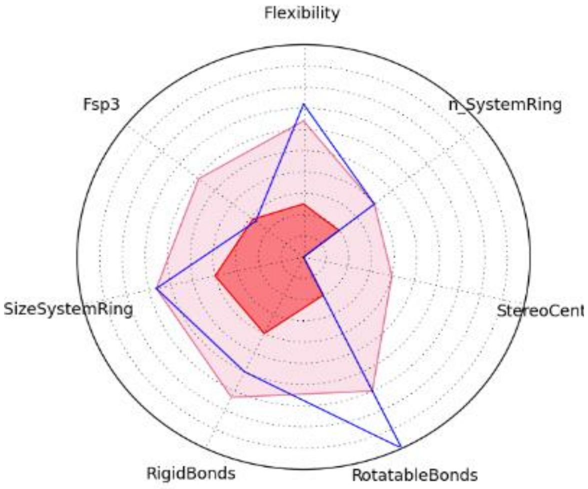

Erlotinib

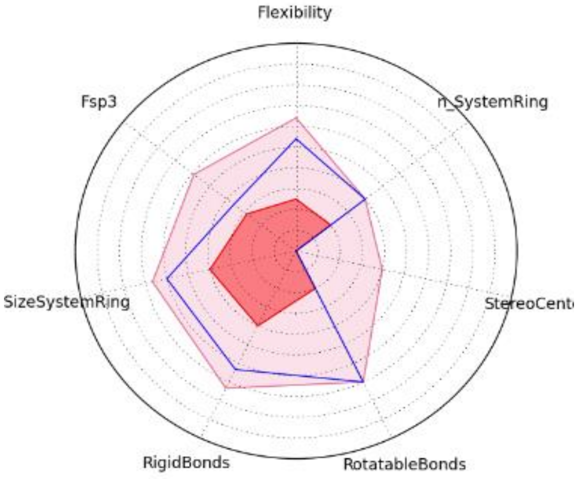

Sunitinib

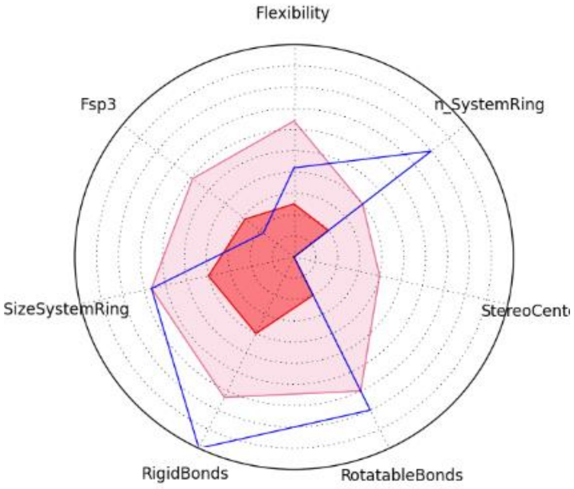

Cabozantinib S-malate

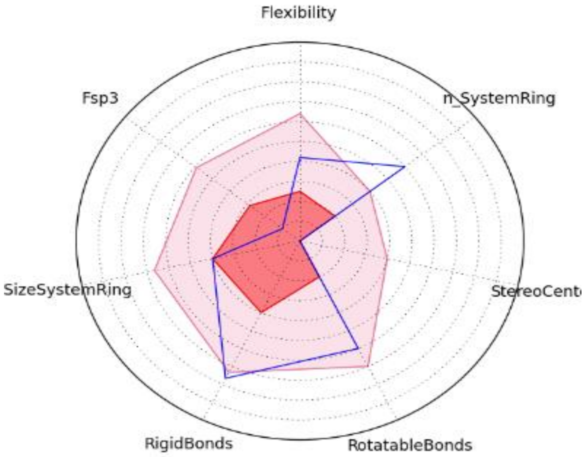

Sorafenib

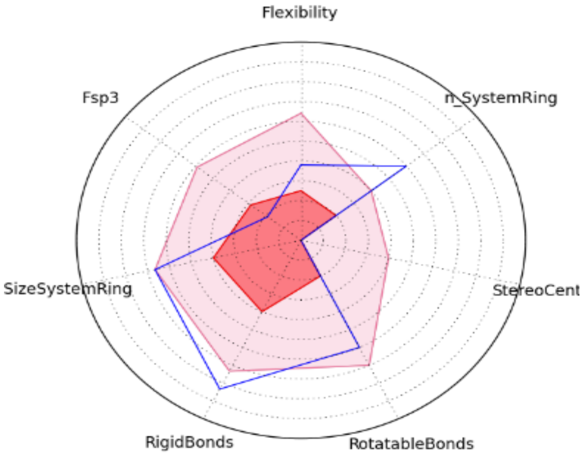

Lenvatinib mesylate

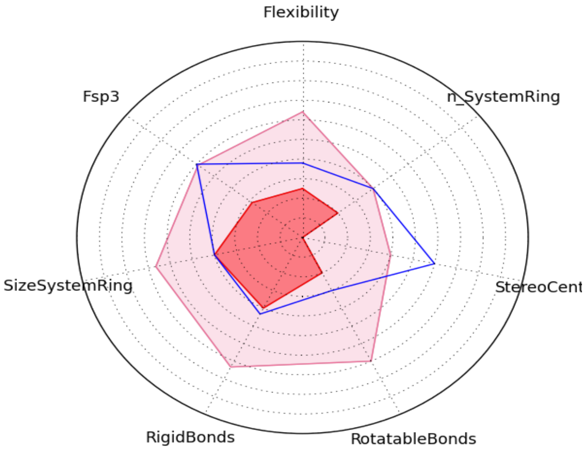

Trifluridine

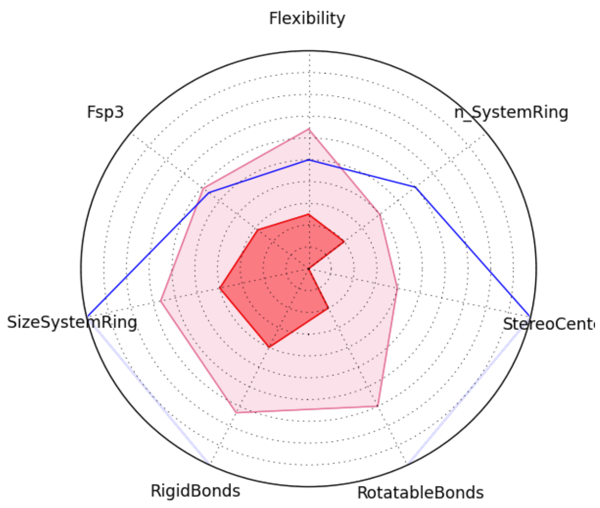

Docetaxel

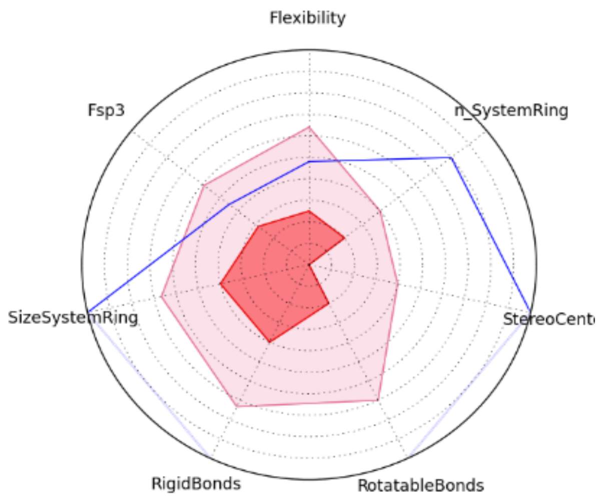

Paclitaxel

Figure S3. Golden Triangle plots for permeability and metabolic stability of the investigated approved anticancer drugs generated with FAF-Drugs4. Each panel corresponds to one investigated drug. Compounds positioned within the triangle are predicted to have a more favorable balance between permeability and metabolic stability, whereas compounds located outside the optimal region may show reduced passive permeability or less favorable clearance-related characteristics.

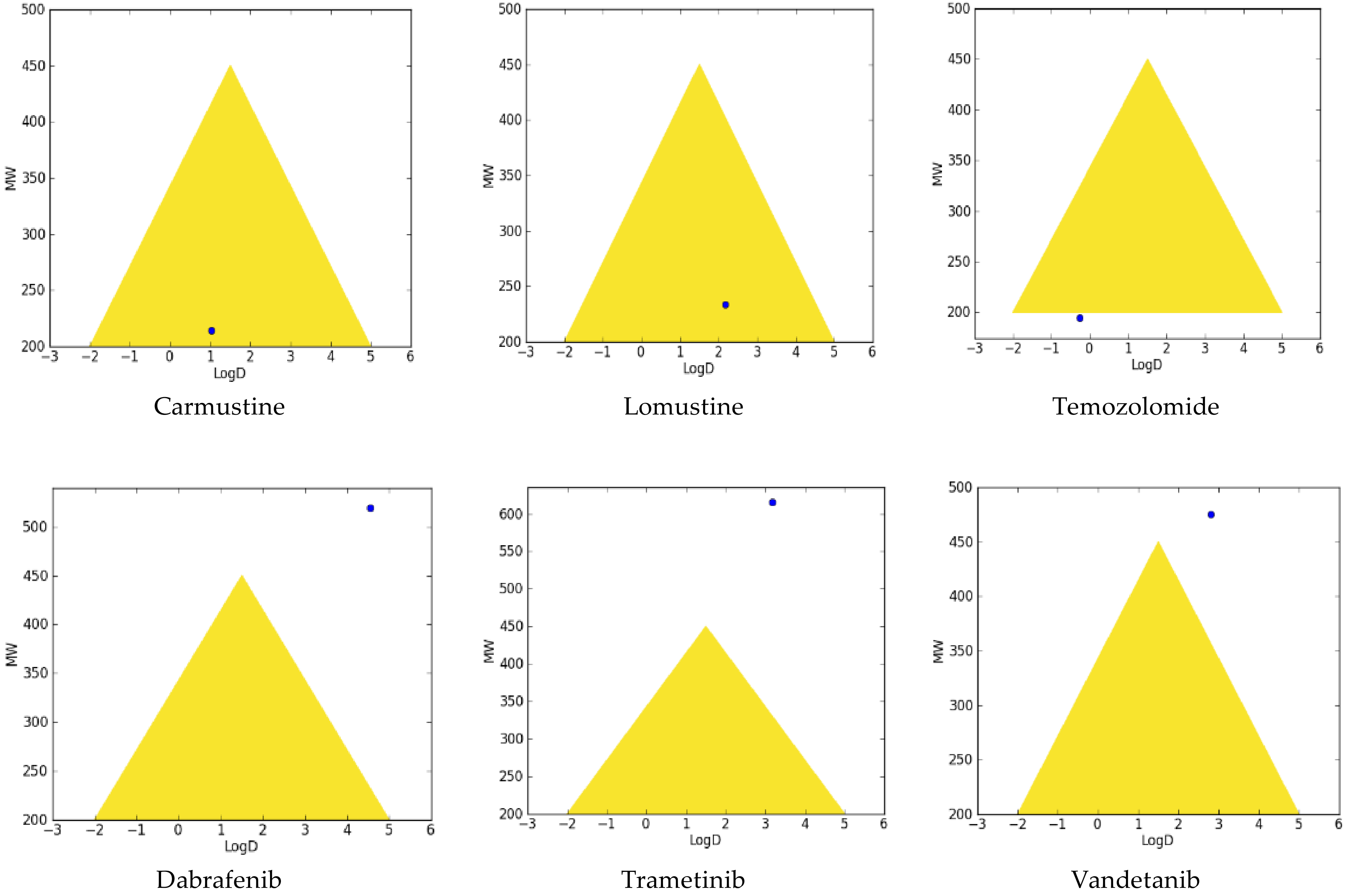

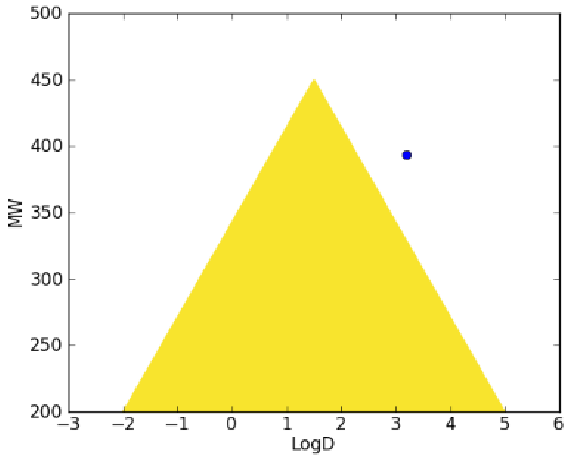

Erlotinib

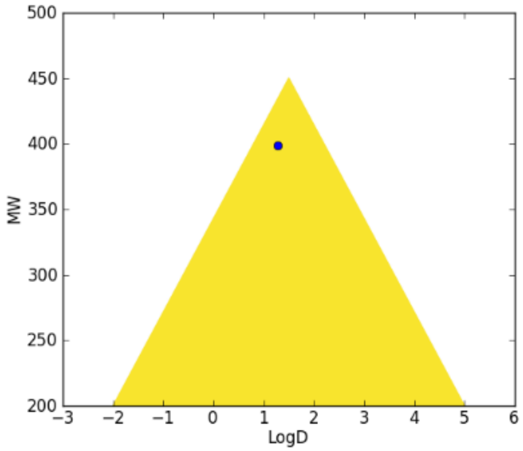

Sunitinib

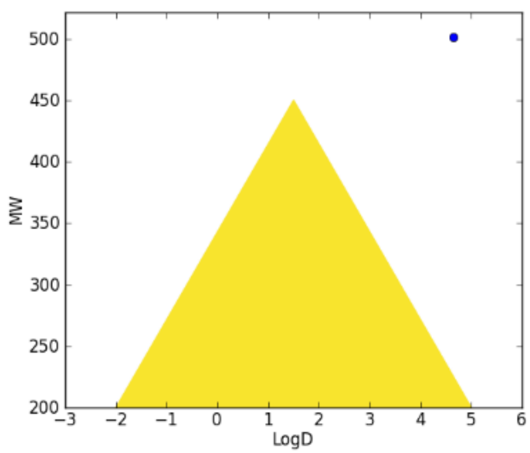

Cabozantinib S-malate

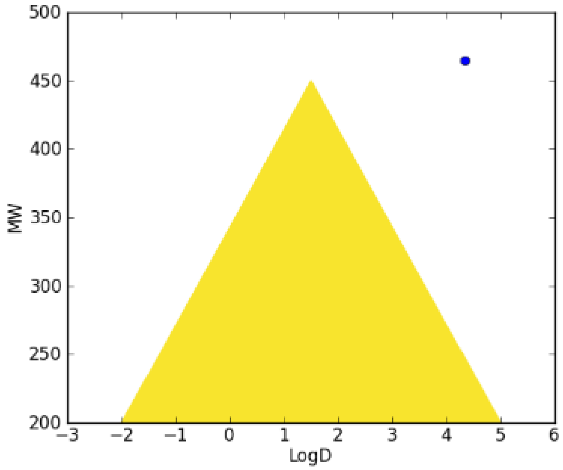

Sorafenib

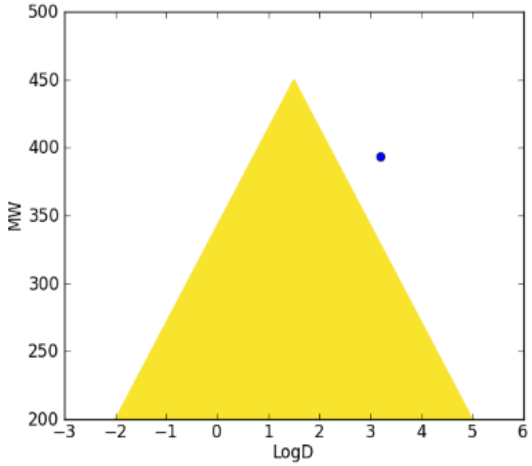

Lenvatinib mesylate

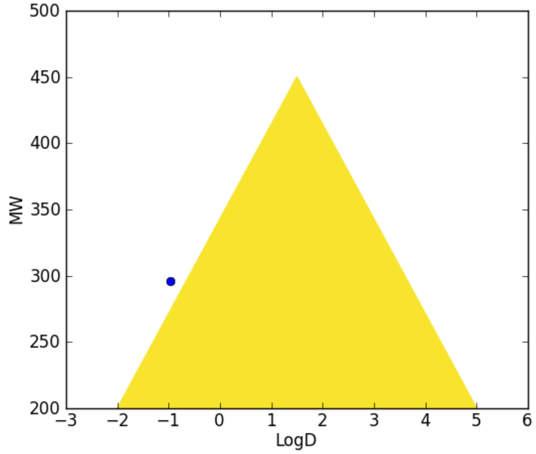

Trifluridine

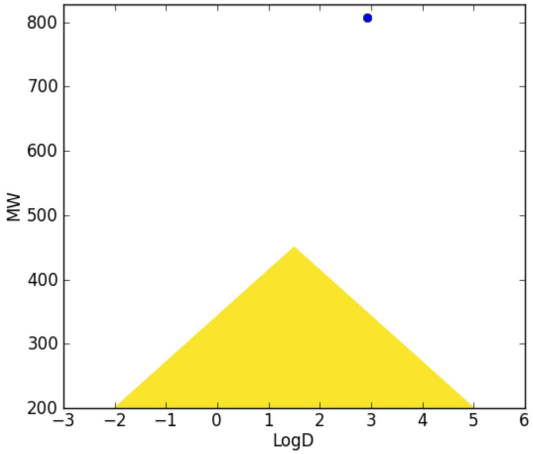

Docetaxel

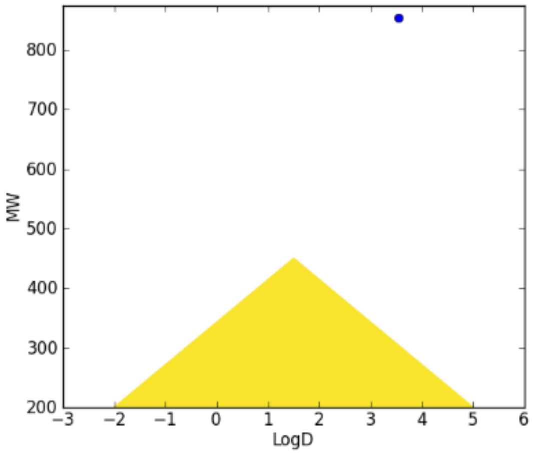

Paclitaxel

Figure S4. Radar plots of the predicted oral absorption profiles of the investigated approved anticancer drugs generated with FAF-Drugs4. Each panel corresponds to one investigated drug. The blue line represents the calculated oral absorption profile of the compound, while the shaded area indicates the physicochemical space generally associated with favorable oral absorption.

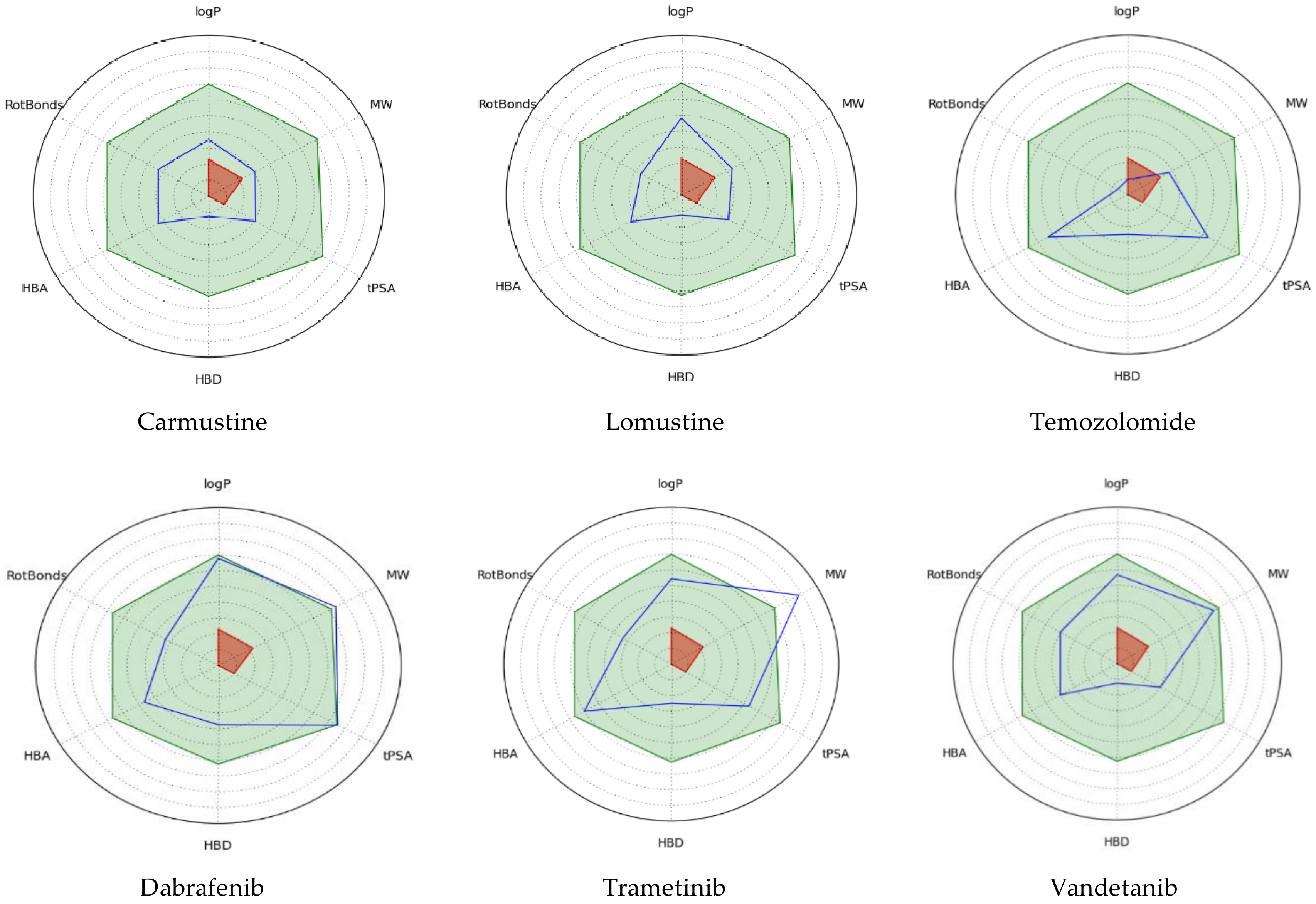

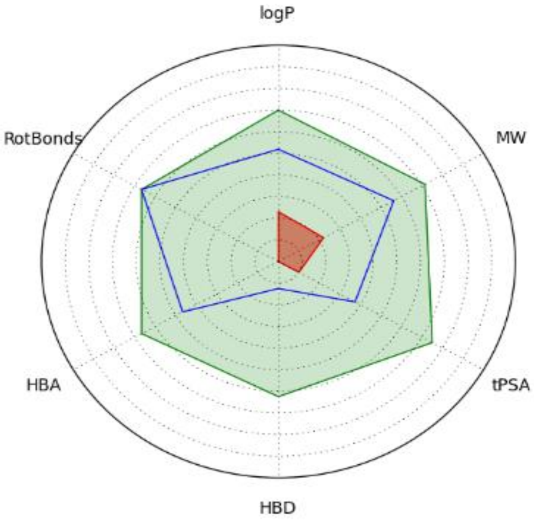

Erlotinib

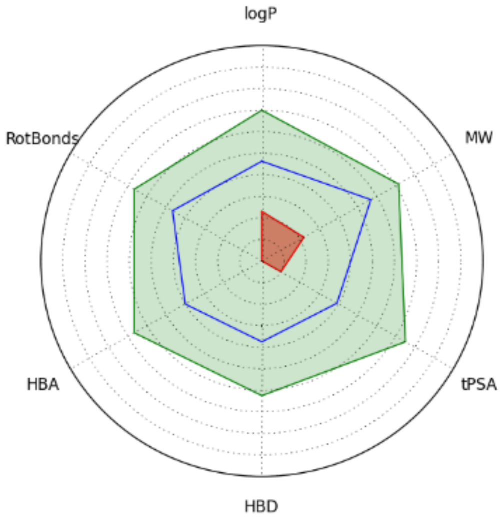

Sunitinib

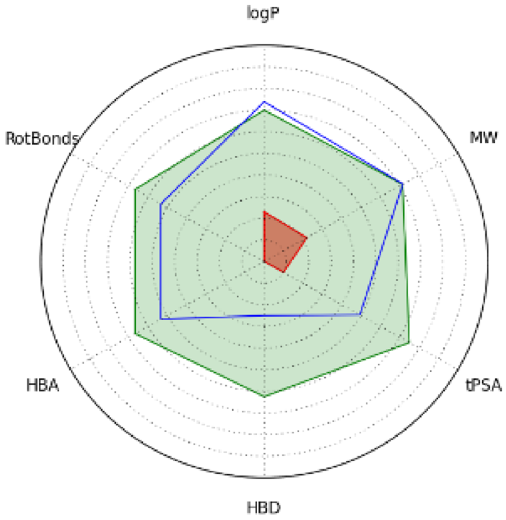

Cabozantinib S-malate

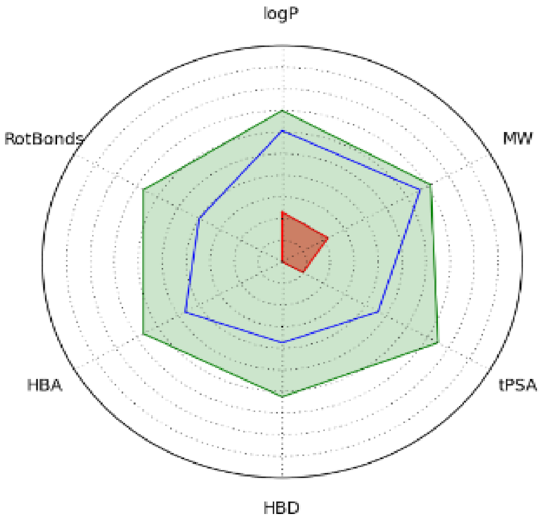

Sorafenib

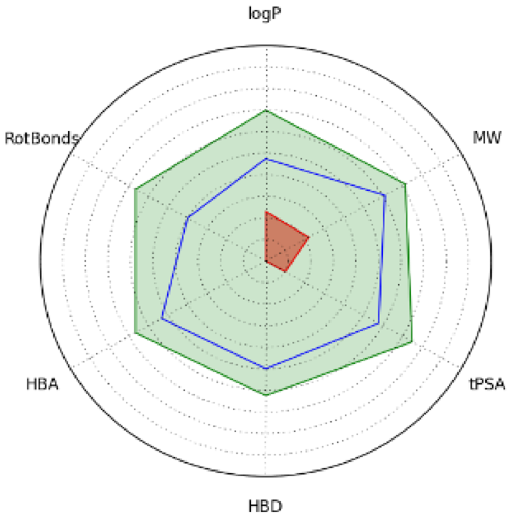

Lenvatinib mesylate

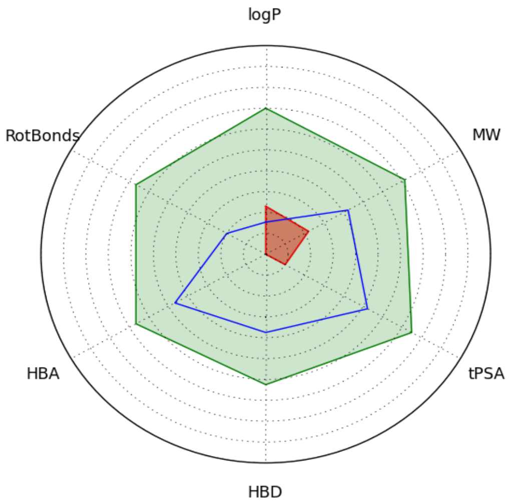

Trifluridine

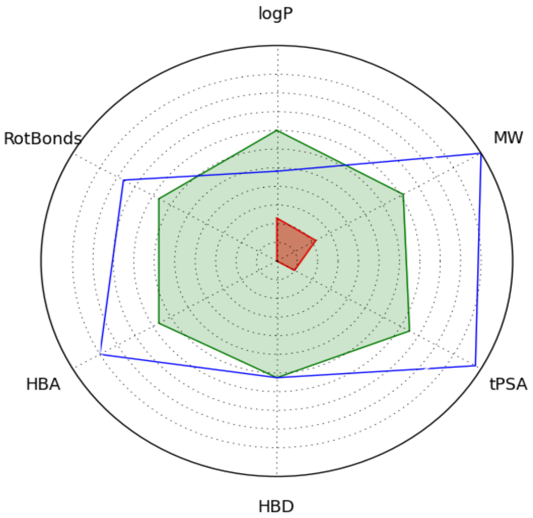

Docetaxel

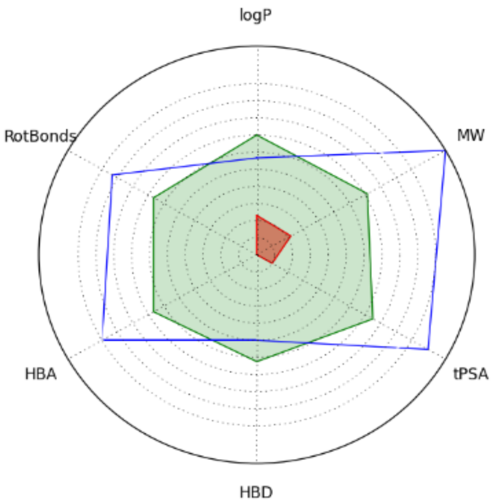

Paclitaxel

Figure S5. Toxicity-space plots of the investigated approved anticancer drugs generated with FAF-Drugs4. Each panel corresponds to one investigated drug. Each blue point represents the position of the compound in the FAF-Drugs4 toxicity-space model according to lipophilicity and topological polar surface area. The dark green region corresponds to the non-toxic space, the light green regions indicate lower predicted toxicity, and the red region represents higher predicted toxicity risk.

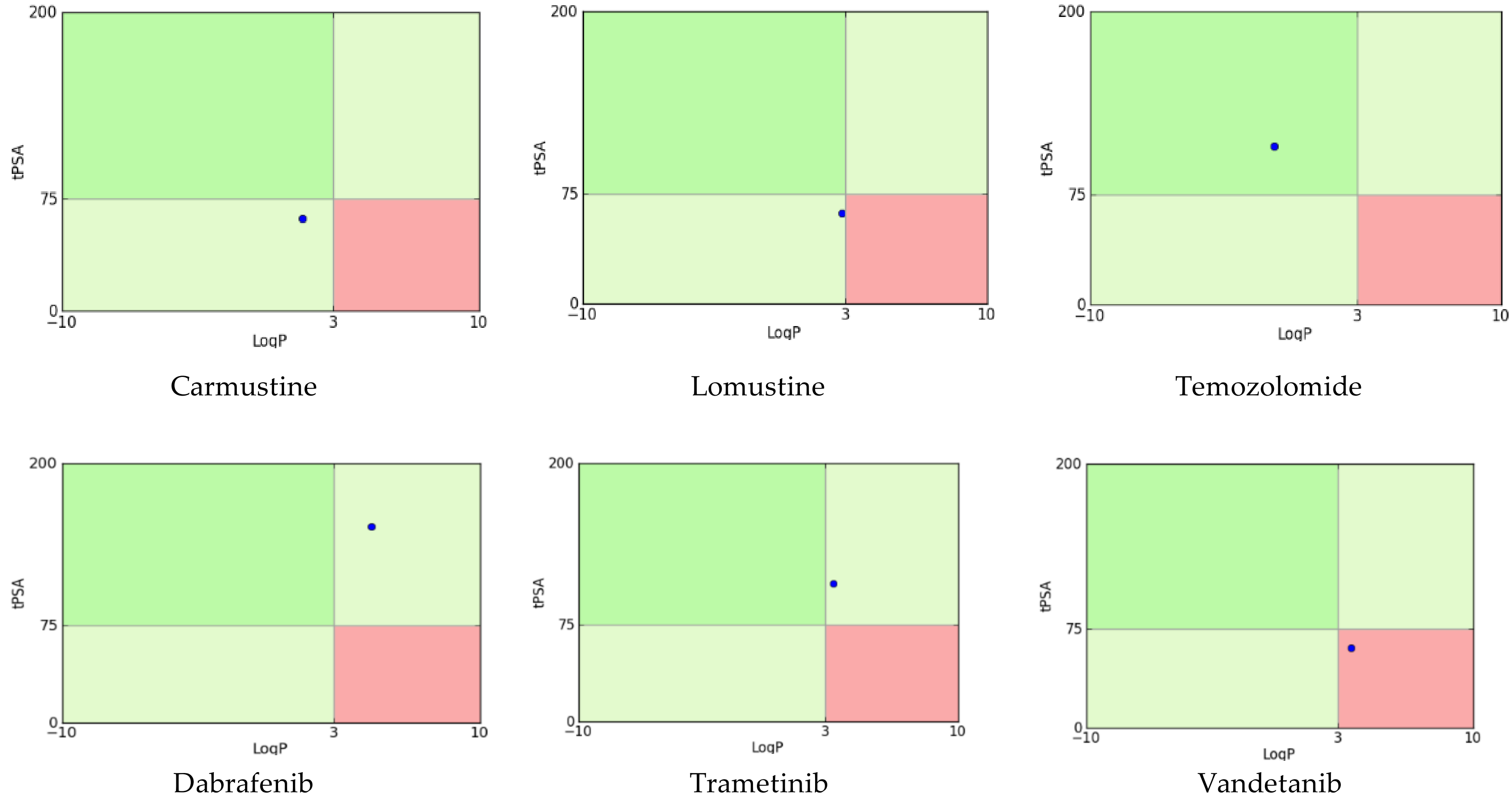

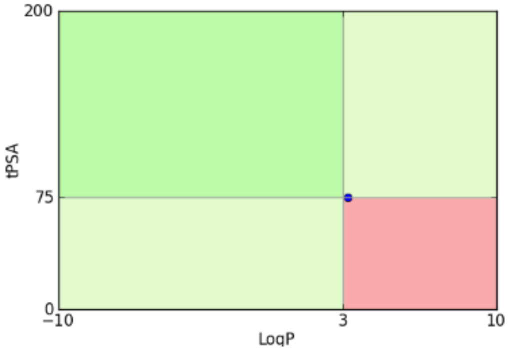

Erlotinib

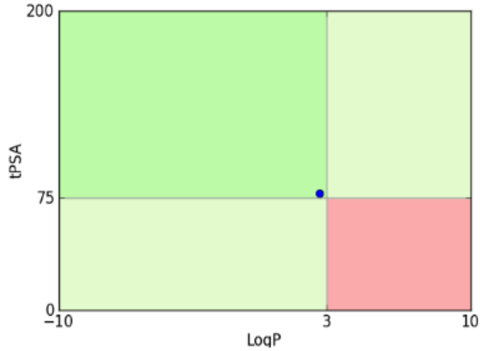

Sunitinib

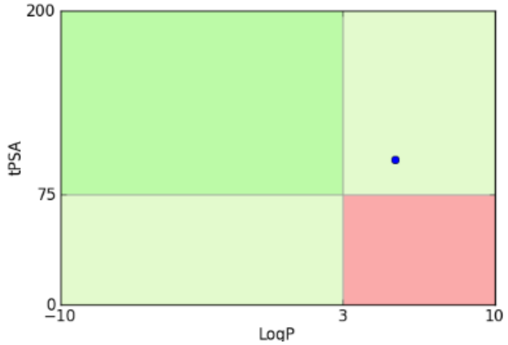

Cabozantinib S-malate

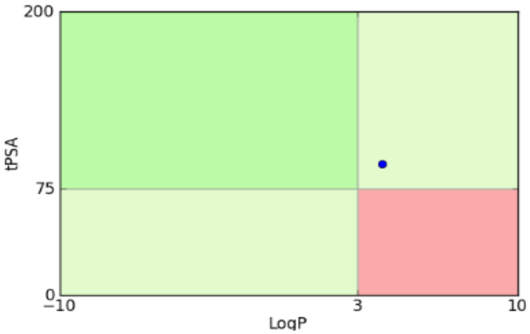

Sorafenib

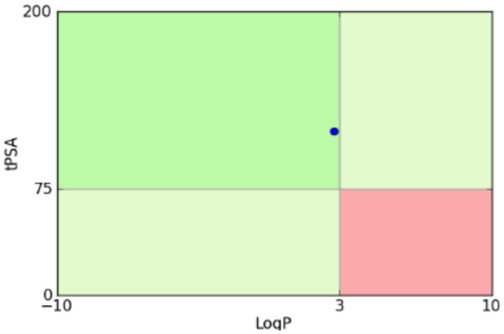

Lenvatinib mesylate

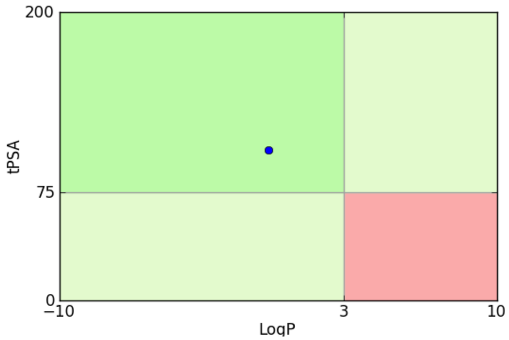

Trifluridine

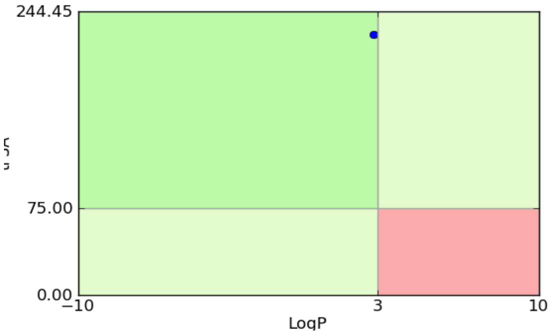

Docetaxel

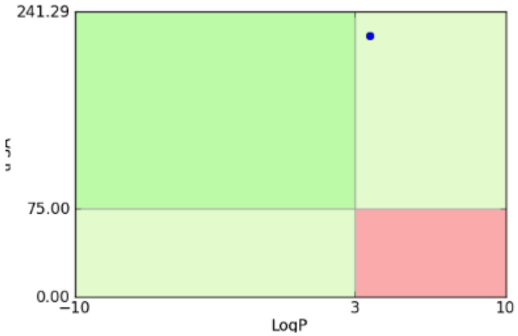

Paclitaxel

Figure S6. BOILED-Egg plots of the investigated approved anticancer drugs generated with SwissADME. Each panel corresponds to one investigated drug. The white region indicates a higher probability of passive gastrointestinal absorption, whereas the yellow yolk indicates a higher probability of blood–brain barrier permeation. Dot color reflects P-glycoprotein substrate status, with blue dots corresponding to predicted P-gp substrates and red dots corresponding to predicted P-gp non-substrates.

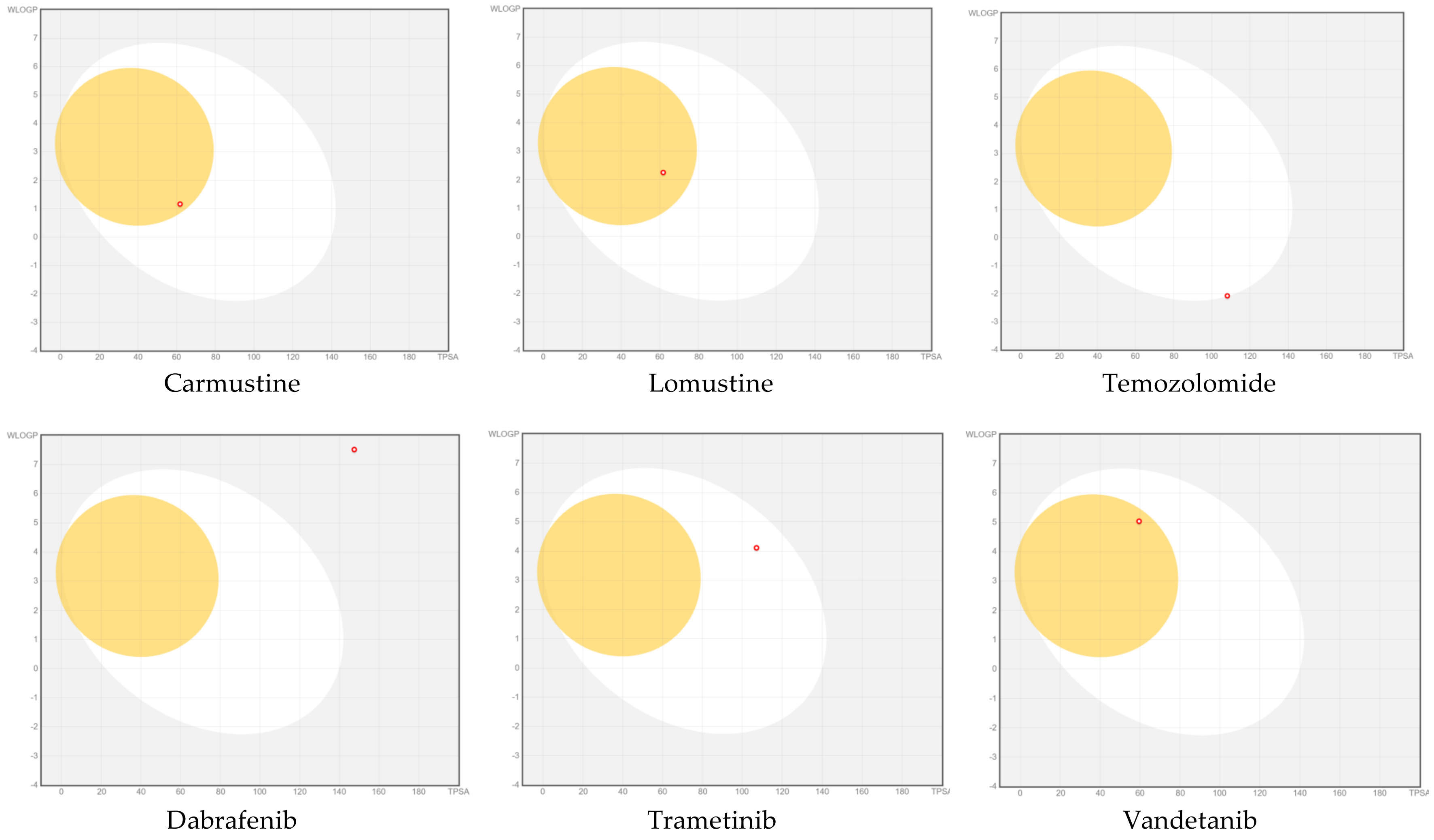

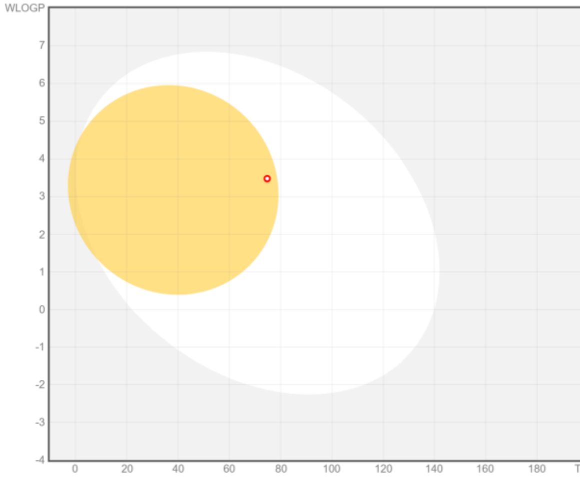

Erlotinib

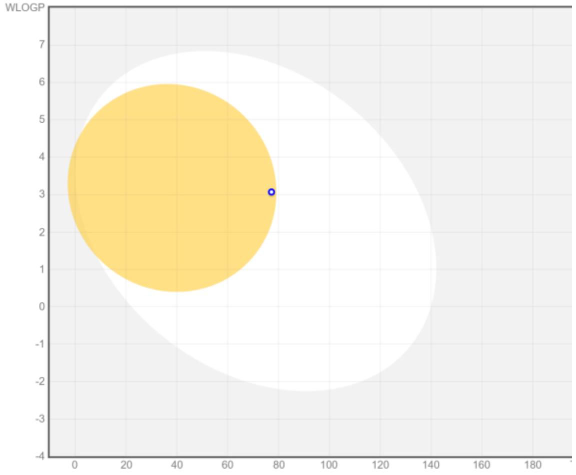

Sunitinib

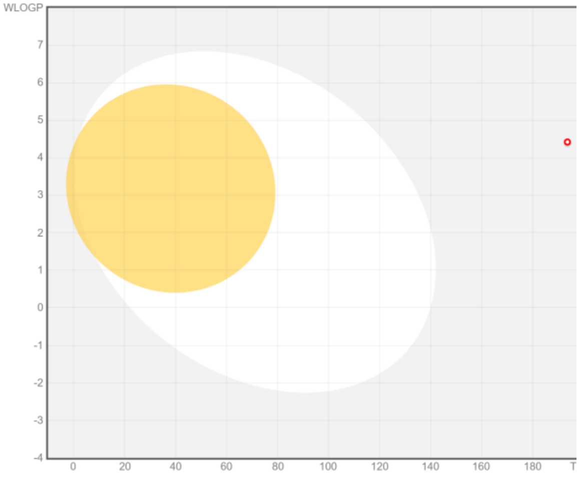

Cabozantinib S-malate

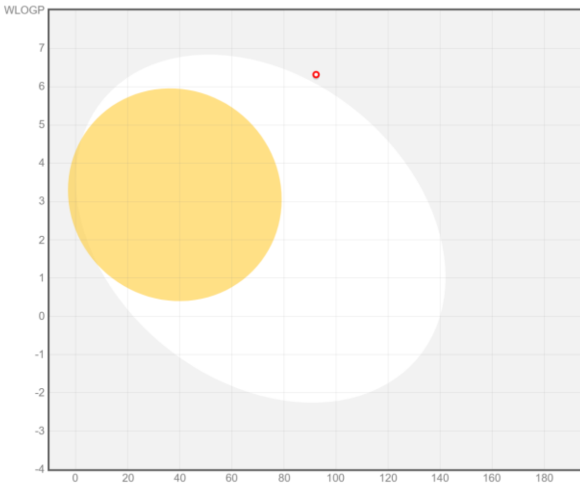

Sorafenib

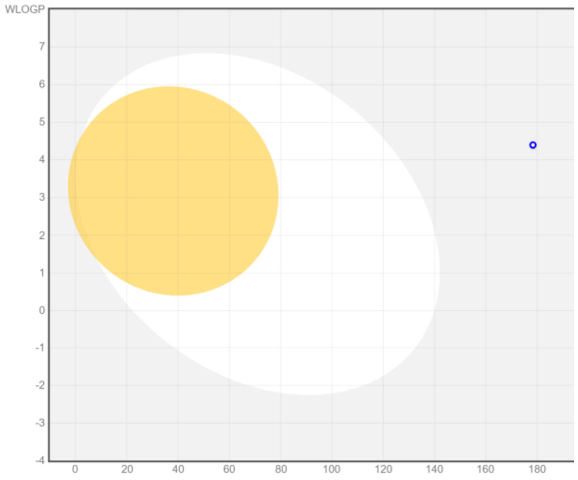

Lenvatinib mesylate

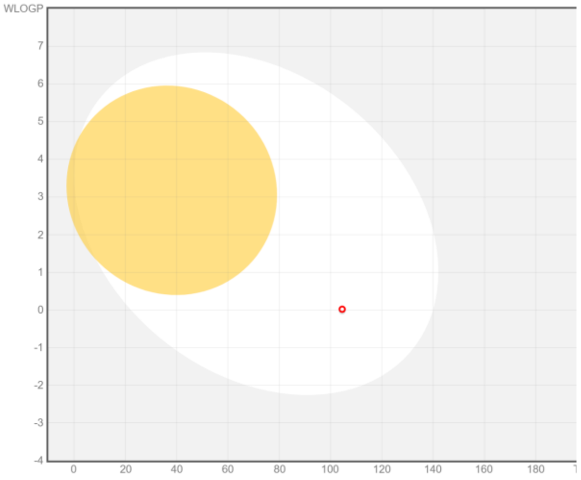

Trifluridine

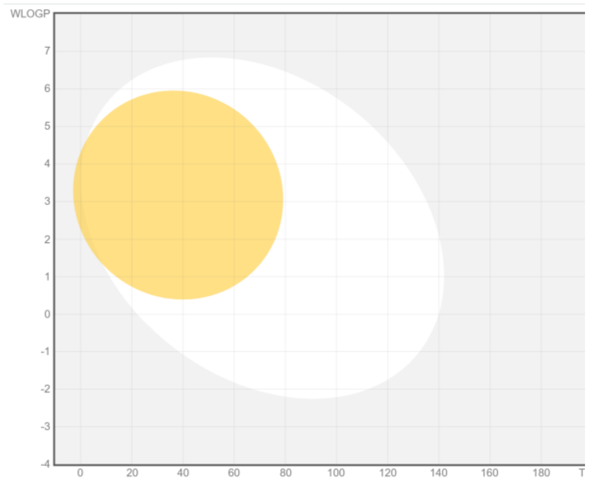

Docetaxel

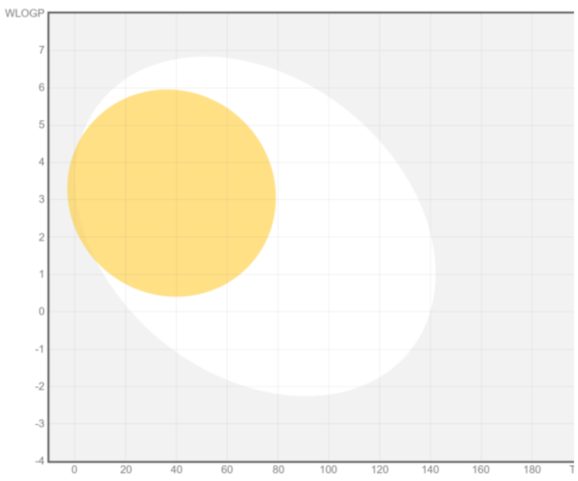

Paclitaxel
